# Unveiling the potential effects of acetylsalicylic acid: insights into regeneration in endometrial stem cells

**DOI:** 10.1186/s12964-023-01339-2

**Published:** 2023-11-10

**Authors:** Se-Ra Park, Soo-Rim Kim, Eun-Kyung Min, Byung-Chul Oh, YunJae Jung, Yong Ho Kim, Hwa-Yong Lee

**Affiliations:** 1 Department of Health Sciences and Technology, GAIHST, Gachon University, Incheon, 21999 Republic of Korea; 2 Department of Molecular Medicine, School of Medicine, Gachon University, Incheon, 406-840 Republic of Korea; 3 Department of Physiology, Lee Gil Ya Cancer and Diabetes Institute, Gachon University College of Medicine, Incheon, 21999 Republic of Korea; 4 Department of Microbiology, College of Medicine, Gachon University, Incheon, 21999 Korea; 5 Gachon Pain Center and Department of Physiology, College of Medicine, Gachon University, Incheon, 21999 Republic of Korea; 6 Division of Science Education, Kangwon National University, 24341 Chuncheon, Republic of Korea

**Keywords:** endometrial stem cells, Acetylsalicylic acid, Regulation, SERPINB2, Self-renewal, Migration, Differentiation

## Abstract

**Background:**

Although acetylsalicylic acid has been widely used for decades to treat and prevent various diseases, its potential effects on endometrial receptivity and subsequent pregnancy rates are still controversial due to conflicting data: many reports have shown positive effects of acetylsalicylic acid, whereas others have found that it has no effect. Furthermore, the direct effects of acetylsalicylic acid on various functions of normal endometrial cells, especially endometrial stem cells, and their underlying molecular mechanisms have not yet been proven. Recently, studies have revealed that a reduced number of active stem/progenitor cells within endometrial tissue limits cyclic endometrial regeneration and subsequently decreases pregnancy success rates, suggesting that endometrial stem cells play a critical role in endometrial regeneration and subsequent endometrial receptivity.

**Methods:**

We assessed whether aspirin treatment can inhibit various endometrial stem cell functions related to regenerative capacity, such as self-renewal, migration, pluripotency/stemness, and differentiation capacity, in vitro*.* Next, we evaluated whether SERPINB2 regulates the effects of aspirin on endometrial stem cell functions by depleting SERPINB2 expression with specific shRNA targeting SERPINB2. To further investigate whether aspirin also inhibits various endometrial stem cell functions in vivo, aspirin was administered daily to mice through intraperitoneal (i.p.) injection for 7 days.

**Results:**

In addition to its previously identified roles, to the best of our knowledge, we found for the first time that acetylsalicylic acid directly inhibits various human endometrial stem cell functions related to regenerative capacity (i.e., self-renewal, migration, differentiation, and capacity) through its novel target gene SERPINB2 in vitro. Acetylsalicylic acid exerts its function by suppressing well-known prosurvival pathways, such as Akt and/or ERK1/2 signaling, through a SERPINB2 signaling cascade. Moreover, we also found that acetylsalicylic acid markedly inhibits regenerative capacity-related functions in endometrial stem cells within tissue.

**Conclusions:**

We have found that acetylsalicylic acid has diverse effects on various endometrial stem cell functions related to regenerative capacity. Our findings are a critical step toward the development of more effective therapeutic strategies to increase the chances of successful pregnancy.

Video Abstract

**Supplementary Information:**

The online version contains supplementary material available at 10.1186/s12964-023-01339-2.

## Introduction

Acetylsalicylic acid effectively inhibits platelet cyclooxygenases (COX-1 and 2) by irreversibly acetylating its active site (serine 530), thus blocking the formation of thromboxane A_2_ [1], which in turn reduces platelet aggregation and subsequently enhances vasodilation. By these indirect mechanisms, acetylsalicylic acid may improve blood circulation in the uterine artery [2], and thus, some clinical researchers have recently tried to improve endometrial receptivity and subsequent implantation with consecutive acetylsalicylic acid treatment. However, there is ongoing controversy regarding its potential effects on endometrial receptivity and pregnancy success rates. For example, Zhang et al. found that the combined use of heparin and acetylsalicylic acid significantly reduces the miscarriage rate [3]. In addition, low-dose acetylsalicylic acid therapy may delay or prevent the occurrence of preeclampsia during pregnancy [4]. Chen et al. also showed that acetylsalicylic acid may increase endometrial thickness and regenerate endometrial damage after surgery for severe intrauterine adhesion [5]. In contrast to these positive outcomes, meta-analyses, and randomized controlled trials (RCTs) regarding the use of low-dose acetylsalicylic acid in women undergoing in vitro fertilization (IVF) therapy to improve endometrial receptivity have shown controversial and ambiguous results [6, 7]. In addition, several studies focusing on the pregnancy outcomes of IVF patients have also obtained conflicting results [8]. Similarly, the findings of Liping et al. suggest that there is no significant improvement in endometrial thickness or implantation rate with the use of acetylsalicylic acid treatment compared to control group [9]. These conflicting results suggest that the impact of acetylsalicylic acid therapy on endometrial receptivity remains a subject of debate.

The endometrium is a highly regenerative tissue that exhibits remarkable cyclic change from 7 to 14 mm during each cycle and is shed in the absence of implantation [10]. Importantly, tissue-resident endometrial stem cells are responsible for the cyclic turnover of the endometrial layer in which the implantation takes place [11]. Indeed, the continuous activation and recruitment of endometrial stem cells to sites of regeneration are necessary for achieving successful pregnancy [12]. A recent study revealed that a decreased number of viable tissue-resident stem cells significantly decreases endometrial receptivity and thus reduces pregnancy rates in patients with recurrent early pregnancy loss (RPL) [12]. According to Lucas et al., a decreased population of clonogenic tissue-resident stem cells within the endometrium can lead to a chronic inflammatory response, potentially resulting in an increased incidence of RPL [13]. Therefore, we have devoted our efforts to investigating whether acetylsalicylic acid has direct effects on various regenerative functions of endometrial stem cells, which play a critical role in endometrial growth and subsequent pregnancy outcomes, and thus affects endometrial receptivity.

Importantly, we found for the first time that acetylsalicylic acid acts as a possible inhibitory factor of endometrial receptivity in that its application significantly inhibits various endometrial stem cell functions related to regenerative capacity, such as proliferative, migratory, and multilineage differentiation potential, in vitro and in vivo. In addition, we also found that acetylsalicylic acid, through its target gene SERPINB2 (also known as plasminogen activator inhibitor type 2), suppresses key pro-survival signaling, including ERK1/2 or Akt pathway, which are involved in essential stem cell functions, such as self-renewal capacity [14], recruitment [15], pluripotency [16], in many stem cell types. Indeed, SERPINB2 knockdown with specific shRNA markedly attenuated the acetylsalicylic acid-mediated inhibitory effects. Moreover, the activation of the PI3K/Akt or FAK/ERK1/2 pathways with selective activators significantly abolished the acetylsalicylic acid-mediated suppressive effects on endometrial stem cells. These results suggest that, in contrast to its previously reported beneficial effects on endometrial receptivity (e.g., enhancing uterine blood flow and tissue perfusion), acetylsalicylic acid suppresses tissue regeneration-associated functions of endometrial stem cells in vitro and in vivo as a possible inhibitory factor of endometrial receptivity.

## Materials and methods

### Isolation and culture of human endometrial stem cells

Human endometrial stem cells were obtained with written informed consent from uterine fibroid patients and approved by the Gachon University Institutional Review Board (IRB No: GAIRB2018-134). The endometrial tissue was minced and digested in DMEM containing 10% FBS and 250 U/ml type I collagenase for 5 h at 37 °C in a rotating shaker, according to a previously established procedure [17]. The resulting mixture was filtered through a 40 µm cell strainer to separate stromal-like stem cells from epithelial gland fragments and undigested tissue. Endometrial stem cells were isolated from other cell types using a single-density Percoll layer by centrifugation for 20 min at 1200 g. The isolated cells were washed twice in PBS and cultured in growth media consisting of various growth factors, including IGF, VEGF, EGF, basic FGF, hydrocortisone, ascorbic acid, heparin, and 10% FBS (Gibco BRL) at 37 °C in a humidified atmosphere of 5% CO_2_ in air. After 3 days, colony-forming cells were isolated using cloning rings (Sigma-Aldrich).

### Isolation and culture of mouse uterine tissue-derived stem cells

The isolation of stem cells derived from mouse uterine tissue was approved and conducted in accordance with the Institutional Animal Care and Use Committee (IACUC) (LCDI-2019–0169) of Gachon University. Uterine tissue was minced into small pieces and digested in DMEM containing 10% FBS and 250 U/ml type I collagenase for 5 h at 37 °C. The resulting mixture was filtered through a 40 µm cell strainer, and the isolated cells were cultured in EBM-2 medium (Lonza) with EGM-2 supplements at 37 °C and 5% CO_2_.

### Cell proliferation assay

To determine the anti-proliferative capacity of acetylsalicylic acid, the MTT assay was performed following the manufacturer's protocol. Endometrial stem cells (2 × 10^4^ cells/well) were seeded in 96-well plates and incubated for 24 h. The cells were then treated with increasing concentrations of acetylsalicylic acid for 48 h. The viable cells were measured at a wavelength of 570 nm using a VersaMax microplate reader.

### Protein isolation and western blot analysis

We analyzed protein expression levels using western blotting as previously described in our studies [17]. We lysed cells in a buffer containing 50 mM Tris, 5 mM EDTA, 150 mM NaCl, 1 mM DTT, 0.01% NP 40, and 0.2 mM PMSF, and measured the protein concentrations of the total cell lysates using bovine serum albumin as a standard. We separated samples containing equal amounts of protein using sodium dodecyl sulfate–polyacrylamide gel electrophoresis (SDS-PAGE), transferred them onto polyvinylidene difluoride (PVDF) membranes (Bio-Rad Laboratories), and blocked the membranes with 5% skim milk in Tris-buffered saline containing Tween-20 at room temperature. We then incubated the membranes overnight at 4 °C with primary antibodies against MMP-2 (Cell Signaling #4022), MMP-9 (Cell Signaling #13,667), SERPINB2 (Abcam, MA, USA, ab47742), total PI3K (Cell Signaling #4292), phospho-PI3K (Cell Signaling #4228), total Akt (Cell Signaling #4491), phospho-Akt (Cell Signaling #4060), total-ERK1/2 (Cell Signaling #9012), phospho-ERK1/2 (Cell Signaling #9101), total FAK (Santa Cruz, sc-558), phospho-FAK (Santa Cruz, sc-11765), or β-actin (Abcam, ab189073), and subsequently incubated them with polyclonal HRP-conjugated goat anti-mouse IgG (BD Pharmingen, 554,002) or goat anti-rabbit IgG (BD Pharmingen, San Diego, CA, USA, 554,021) secondary antibodies at room temperature for 60 min. Finally, we detected the antigen–antibody complexes using Western blot ECL reagents (GE Healthcare, Bucks, UK).

### In vitro cell migration assay

We assessed the impact of acetylsalicylic acid on the migration ability of endometrial stem cells by calculating the ratio of cells that migrated in response to acetylsalicylic acid treatment versus the number of cells that migrated spontaneously. To track cell migration, we plated cells at a density of 1 × 10^5^ cells/well in 200 μL of culture medium in the upper chambers of permeable Transwell supports (Corning Inc., Corning, NY, USA). These Transwell chambers had 8.0 μm pores in 6.5-mm diameter polycarbonate membranes and were arranged in a 24-well plate format. After incubation, non-migrated cells on the upper surface of each membrane were removed by scrubbing with laboratory paper. Migrated cells on the lower surface of each membrane were then fixed with 4% paraformaldehyde for 5 min and stained with hematoxylin for 15 min. Finally, we counted the number of migrated cells in three randomly selected fields of each well using a light microscope at 50X magnification. The difference between the groups was expressed as a fold change.

### Real-time PCR analysis

Total RNA was extracted from human or mouse endometrial stem cells using commercial TRIzol® reagent (Invitrogen Life Technologies, CA, USA) according to the manufacturer’s recommended instructions. RNA purity was estimated by measuring the ratio of absorbance at 260 nm and 280 nm. The first-strand cDNA was synthesized using SuperScript II Reverse Transcriptase (Invitrogen Life Technologies, CA, USA) with 1 μg of total RNA. The first-strand cDNA was synthesized using Express SYBR-Green qPCR Supermix (BioPrince, Seoul, South Korea). qPCR was performed using a QIAGEN's real-time PCR cycler, the Rotor-Gene Q. The relative mRNA expression levels of the target genes were calculated as fold changes using the ΔΔCT method. The sequences of the PCR primers are listed in Table 1.

**Table 1:** Primer sequences for quantitative RT-PCR

**Primer sequence**			
TGCCATCGCCAAGGAGTAG			
TGCACAGACGGTCACTCAAA			
AAAGGCCCCCAAGGTAGTTA			
GCACAAGAGTTCCGTAGCTG			
TGGGATTTACAGGCGTGAGC			
AAGCAAAGCCTCCCAATCCC			
AGCCCTCATTTCACCAGGCC			
TGGGACTCCTCCGGGTTTTG			
AAATGGGAGGGGTGCAAAAGAGGAG			
CAGCTGTCATTTGCTGTGGGTGATG			
ACCCCCATGACTCCAGAGAACT			
GAGAGCGGAAGGATGAATGGAT	NM_003106	F	AAATGGGAGGGGTGCAAAAGAGGAG
GCCTAAGATGAGCGCAAGTTG		R	CAGCTGTCATTTGCTGTGGGTGATG
TACTAGGCAGATGGCCACAGG	01143818	F	ACCCCCATGACTCCAGAGAACT
CGCACACACAACGTCTTGGA	NM_003106	F	AAATGGGAGGGGTGCAAAAGAGGAG
AGGATGTAGGCGGTGGCTTT		R	CAGCTGTCATTTGCTGTGGGTGATG
GCCTTACGTACAGTTGCAGC	NM_001143818	F	ACCCCCATGACTCCAGAGAACT
GCCTTACGTACAGTTGCAGC			
GCATTCAAACTGAGGCACCA			
AGCTTCTTTCCCCATCCCA			
GAAGCGTGTACTTATCCTTCTTCAT			
GAGTGGAAACTTTTGTCCGAGA	NM_011443	F	GAAGCGTGTACTTATCCTTCTTCAT
ACTTAATGGGCTTTATCCTTTCC	NM_011443	F	GAAGCGTGTACTTATCCTTCTTCAT
TGCGTCCTCAATCTCATCG		R	GAGTGGAAACTTTTGTCCGAGA

### Ingenuity pathway analysis

The Ingenuity Pathway Analysis (IPA) version 2.0 software (Ingenuity Systems, Redwood City, CA) was used to analyze the genes related to SERPINB2. Genes that were differentially expressed between proliferative and non-proliferative cells (t-test, *P* < 0.005) were subjected to analysis of EGFR (GSE21618), IGFBP6 (GSE47856), M-CSF (GSE45630), PDGFRB (GSE116237), SCFR (GSE46045), or TGFß2 (GSE48990)-related genes. Fisher's exact test (*P* value) was used to measure the significance of each molecule, identifying differentially expressed genes from the microarray data that overlapped with known regulated genes. The activation score (z score) was used to indicate the predicted molecule status by comparing the observed differential regulation of genes ("up" or "down") in the microarray data relative to the literature-derived regulation direction, which can either activate or inhibit.

### Flow cytometry

Flow cytometry analysis and cell sorting were conducted using FACS Calibur and FACS Aria machines (Becton Dickinson, Palo Alto, CA), respectively. Flow cytometry data were analyzed with FlowJo software (Tree Star, Ashland, OR). The antibodies used for detection included PE-conjugated CD34 (MACS; Miltenyi Biotech, 30–081-002, dilution 1/40), CD44 (MACS; Miltenyi Biotech, 130–095-180, dilution 1/40), CD45 (MACS; Miltenyi Biotech, 130–080-201, dilution 1/40), CD73 (MACS; Miltenyi Biotech, 130–095-182, dilution 1/40), CD105 (MACS; Miltenyi Biotech, 130–094-941, dilution 1/40), CD140b (MACS; Miltenyi Biotech, 130–105-279, dilution 1/40), and W5C5 (MACS; Miltenyi Biotech, 130–111-641, dilution 1/40). FACS gates were set using either an isotype antibody or a secondary antibody.

### Analysis of Gene Expression Omnibus (GEO) database

Gene Expression Omnibus (GEO) is an open-access database repository that stores high-throughput gene expression data generated by genome hybridization arrays, chip sequencing, and DNA microarrays [18, 19]. Researchers can upload their experimental results in four categories: experimental designs, sample, platform, and raw data. Within each dataset, clinical or experimental samples are further classified into various experimental subgroups based on treatment, physiologic condition, and disease state. This categorized biological data is presented as a "GEO profile," which includes the dataset title, gene annotation, a chart showing the expression levels and rank for each gene across the samples [20]. To analyze the expression profiles of EGFR, IGFBP6, M-CSF, PDGFRB, SCFR, or TGFß2 in response to various acetylsalicylic acid treatment conditions, we followed previously established procedures [20].

### Adipocyte differentiation

Human or mouse endometrial stem cells were cultured in low-glucose DMEM supplemented with 500 µM methylxanthine, 5 µg/mL insulin, and 10% FBS for 3 weeks with medium change twice per week. The formation of lipid droplets was confirmed by oil red O staining, and their abundance was measured by calculating the absorbance at 500 nm.

### Osteoblast differentiation

Endometrial stem cells derived from either human or mouse were cultured in high-glucose DMEM supplemented with 0.1 µM dexamethasone, 10 mM β-glycerophosphate, 50 µM ascorbate, and 10% FBS for a period of three weeks with medium replacement twice per week. After differentiation, cells were stained with alizarin red S to detect the formation of new bone matrix. To quantify the presence of alizarin red S in the samples, the optical density (OD) of the solution was measured at 570 nm.

### SERPINB2 knockdown

Bioneer (Daejeon, South Korea) provided small hairpin RNA (shRNA: accession No. NM_002575) targeting SERPINB2 and scrambled shRNA (shCon). To achieve efficient SERPINB2 transfection, reverse transfection was conducted using Lipofectamine 2000 (Invitrogen) as per the manufacturer's instructions. Specifically, shRNA targeting SERPINB2 (3 μg/ml) was mixed with 3 μl transfection reagent lipofectamine 2000 in Gibco opti-MEM media without FBS and antibiotics. Five hours before transfection, opti-MEM was replaced with fresh EGM-2 medium containing 10% FBS. The most effective SERPINB2 shRNA, as determined by qRT-PCR analysis, was selected from three shRNA designed from the target sequence.

### Growth factor antibody array

The manufacturer's protocol (Abnova AA0089) was followed to perform the assay. In brief, protein samples treated with acetylsalicylic acid or vehicle were incubated with antibody membranes overnight at 4 °C. After washing with wash buffer for 3 times, biotin-conjugated anti-cytokine antibodies were incubated with the membranes overnight at 4 °C. The membranes were then washed 3 times and incubated with HRP-conjugated streptavidin. Detection of signals of the growth factors that were spotted on the nitrocellulose membrane was performed using chemiluminescence.

### Evaluation of the effects of acetylsalicylic acid treatment in animal model

Even though it does not entirely depict the physiological features of the human body, the outcomes achieved in vitro were validated through the use of mice, which are commonly utilized as in vivo models for evaluating efficacy. Animal experiments were conducted in accordance with the Institutional Animal Care and Use Committee (IACUC) (LCDI-2019–0169) of the Gachon University, and all protocols were approved. All experiments were designed and reported in accordance with the Animal Research: Reporting of In Vivo Experiments (ARRIVE) guidelines. The animals were housed in standard cages with ad libitum access to food (standard chow diet) and water. The animal room was maintained on a 12-h light/dark cycle at a constant temperature of 22 °C. The female mice (C57BL/6) were blindly and randomly divided into two groups (*n* = 5 per group sufficient to determine differences by treatment): control and acetylsalicylic acid treatment (50 mg/kg for 7 consecutive days intravenously). All animals were involved in data analyzing. After anesthesia and exsanguination by cardiac puncture, stem cells were isolated from uterine and adipose tissues. The isolated stem cells from endometrium, adipose tissue, or bone marrow were then cultured and expanded in vitro with continuous exposure to acetylsalicylic acid (2.5 mM) to simulate the physiological conditions of acetylsalicylic acid exposure in vivo. Then, the effects of aspirin on the self-renewal, migration, pluripotency, and differentiation capacity of stem cells in vivo were evaluated.

### Statistical analysis

The experimental data were presented as mean ± standard deviation (SD) and were obtained from a minimum of three independent experiments. Statistical analysis among the experimental groups was performed using GraphPad Prism 5.0 (GraphPad Software, Inc., La Jolla, CA, US) with one-way ANOVA. Significance was set at *p* < 0.05.

## Results

### Acetylsalicylic acid significantly suppresses various endometrial stem cell functions related to regenerative capacity in vitro

**Fig. 1:**
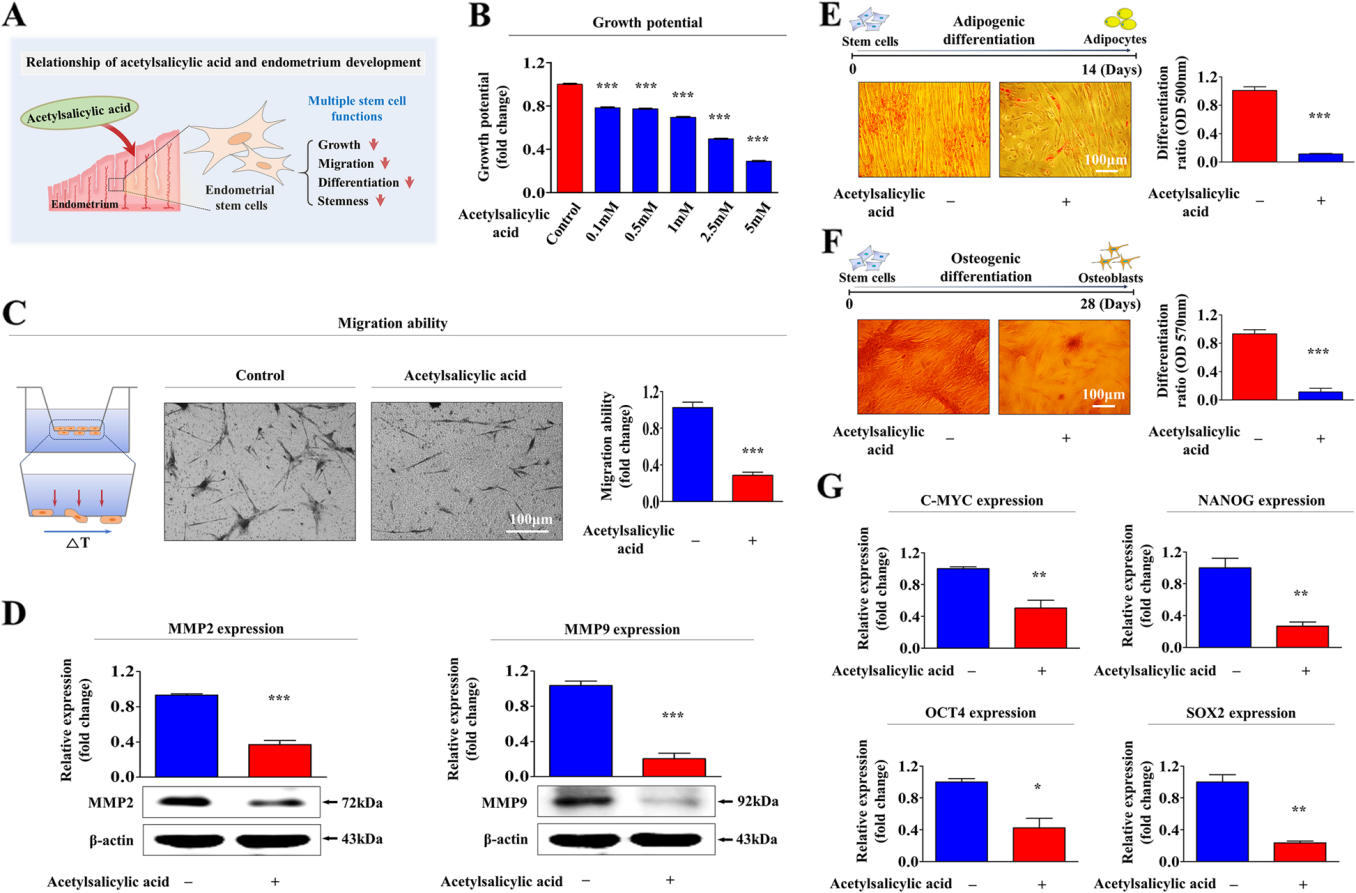
Acetylsalicylic acid treatment markedly suppresses multiple beneficial functions of endometrial stem cells in vitro. We evaluated whether acetylsalicylic acid treatment inhibits various regenerative capacity-associated functions of endometrial stem cells, such as self-renewal, migration, pluripotency and multilineage differentiation, in vitro (**A**). The inhibition of self-renewal capacity by treatment with multiple concentrations of acetylsalicylic acid (100 nM, 500 nM, 1 mM, 2.5 mM, and 5 mM) was analyzed at 48 h using MTT assays. The stem cell proliferation rates (%) were assessed by representing the viability of the acetylsalicylic acid-treated cells as a percentage of the viability of the vehicle-treated cells (**B**). Endometrial stem cells were treated with acetylsalicylic acid (2.5 mM) for 72 h, and the acetylsalicylic acid-induced inhibition of migratory potential was then analyzed by Transwell migration/invasion assays (**C**). The levels of the well-known migration regulatory proteins MMP-2 (72 kDa) and MMP-9 (92 kDa) in cells with or without acetylsalicylic acid treatment were analyzed using western blotting (**D**). Endometrial stem cells were incubated in adipogenic or osteogenic differentiation medium for 2 weeks with or without acetylsalicylic acid (2.5 mM) treatment. The acetylsalicylic acid-induced inhibition of the adipogenesis (**E**) and osteogenesis (**F**) of endometrial stem cells was analyzed using oil red O and alizarin red S staining, respectively. The cytoplasmic calcium concentration and lipid droplet (LD) formation within differentiated cells were assessed by measuring the absorbance values of the solubilized cells at wavelengths of 500 nm and 570 nm, respectively (**E**). The acetylsalicylic acid-induced inhibition of the expression of various pluripotency/stemness markers (C-MYC, NANOG, OCT4, and SOX2) was evaluated using real-time PCR (**G**). β-actin was used as an internal control to normalize protein expression. PPIA was used as a reference gene to normalize gene expression. All experiments were performed in triplicates. Significant differences are presented. *p < 0.05, **p < 0.005, and ***p < 0.001 (One-way ANOVA)

Human endometrial stem cells were isolated from endometrial biopsies according to the method described earlier by Cho et al. [21]. These cells were characterized by analyzing various stem cell surface antigens using flow cytometry (Suppl. Figure 1A-B). Their pluripotency/stemness was also assessed by inducing into adipogenic (Suppl. Figure 1C) and osteogenic (Suppl. Figure 1D) differentiation. A schematic representation of our results showing the novel functions of acetylsalicylic acid is presented in Fig. 1A. We assessed whether acetylsalicylic acid treatment can inhibit various endometrial stem cell functions related to regenerative capacity. The proliferative capacity of endometrial stem cells was markedly reduced by acetylsalicylic acid treatment (Fig. 1B). In addition, acetylsalicylic acid treatment also reduced endometrial stem cell migration (Fig. 1C). To further assess the acetylsalicylic acid-induced inhibitory effects on their migratory capacity, the protein levels of MMP-2 and -9, which induce the proteolytic degradation of the ECM and thus regulate cell migration, were analyzed (Fig. 1D). Interestingly, acetylsalicylic acid treatment significantly decreased their adipogenic (Fig. 1E) and osteogenic (Fig. 1F) differentiation capacities. The expression of major stemness-associated factors, including C-MYC, OCT4, NANOG, and SOX2, was also markedly reduced by acetylsalicylic acid (Fig. 1G). These results indicate that acetylsalicylic acid significantly suppresses various endometrial stem cell functions related to regenerative capacity, such as proliferative, migratory, stemness, and multilineage differentiation potential.

### The suppressive effects of acetylsalicylic acid on the regenerative capacity of endometrial stem cells are mediated through its target gene SERPINB2

**Fig. 2:**
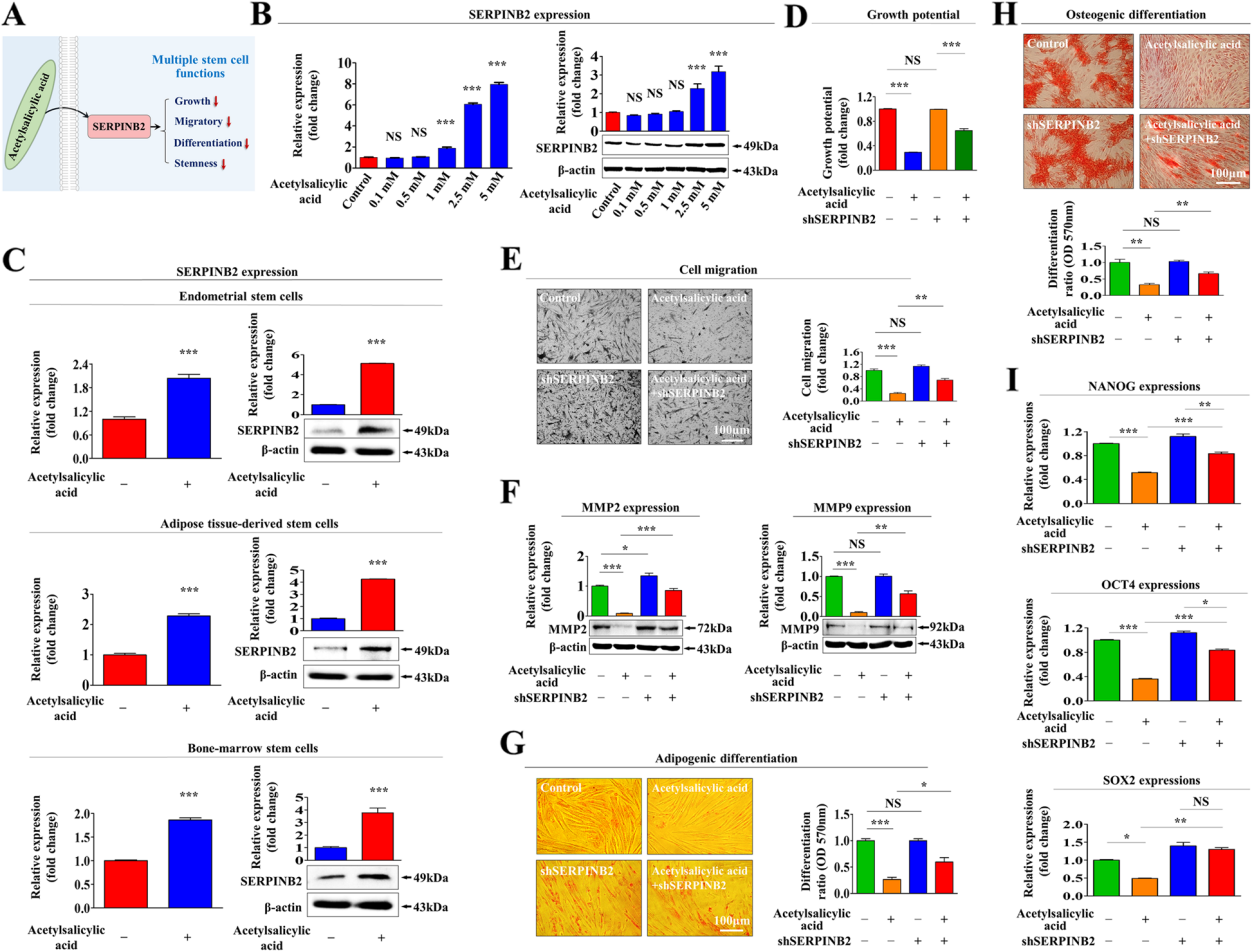
Acetylsalicylic acid exerts diverse effects on the regenerative capacity of endometrial stem cells through its target gene SERPINB2. A schematic representation of the regulatory role of SERPINB2 in mediating the diverse effects induced by acetylsalicylic acid is shown (**A**). Endometrial stem cells were treated with or without acetylsalicylic acid (2.5 mM) for 72 h. The in vitro effects of acetylsalicylic acid treatment on the mRNA and protein expression levels of SERPINB2 in a dose-dependent manner were analyzed using real-time PCR and western blotting, respectively (**B**). In addition, mice were intraperitoneally treated with acetylsalicylic acid (50 mg/kg daily for 7 consecutive days) or vehicle (PBS). Various stem cell types were isolated from the mouse uterine endometrium, bone marrow, and adipose tissues, and the changes in SERPINB2 expression were subsequently analyzed using real-time PCR and western blotting (**C**). Endometrial stem cells were transfected with specific shRNA targeting SERPINB2 and then treated with or without acetylsalicylic acid (2.5 mM); the subsequent changes in self-renewal capacity were analyzed at 48 h using MTT assays (**D**). The effects of SERPINB2 knockdown on the acetylsalicylic acid-induced changes in the migration potential of endometrial stem cells were also assessed by in vitro Transwell assays (**E**) and western blotting with antibodies against MMP-2 and MMP-9 (**F**). The effects of SERPINB2 depletion on the acetylsalicylic acid-induced inhibition of adipogenic (**G**) and osteogenic (**H**) differentiation were evaluated using oil red O staining and alizarin red S staining, respectively. The attenuating effects of SERPINB2 depletion on the acetylsalicylic acid-induced changes in the levels of the pluripotency/stemness-associated transcription factors NANOG, OCT4, and SOX2 were also assessed by real-time PCR (**I**). β-actin was used as an internal control to normalize protein expression. PPIA was used as a reference gene to normalize gene expression. All experiments were performed in triplicates. Significant differences are presented. *p < 0.05, **p < 0.005, and ***p < 0.001 (One-way ANOVA)

Previous studies have found that toxic exposure may induce the premature senescence of resident stem cells in the endometrium [22], leading to adverse effects on endometrial functions [23, 24]. Recently, Part et al. revealed that SERPINB2 is involved in the regulation of various stem cell functions, such as proliferative, migratory capacity, and aging phenotypes [21]. In this context, we therefore determined whether acetylsalicylic acid exerts its inhibitory effects on endometrial stem cells through SERPINB2 (Fig. 2A). Interestingly, our results revealed that acetylsalicylic acid treatment significantly increased SERPINB2 expressions in a dose-dependent manner (Fig. 2B). Consistently, daily intraperitoneal injections of acetylsalicylic acid (50 mg/kg) for 7 days suppressed SERPINB2 expressions in various tissue-derived stem cells, such as adipose tissue, endometrium, bone marrow (Fig. 2C). Thus, we evaluated whether SERPINB2 regulates the function of acetylsalicylic acid in endometrial stem cells by knocking down SERPINB2 expression using its specific shRNA (Suppl. Figure 2A-C). Indeed, the acetylsalicylic acid-mediated suppression of proliferative capacity was significantly attenuated by SERPINB2 depletion (Fig. 2D). The acetylsalicylic acid-induced suppression of migration potential (Fig. 2E) and the expressions of MMP-2 and -9 (Fig. 2F) were also clearly diminished by SERPINB2 knockdown. Importantly, we also observed that the acetylsalicylic acid-mediated suppression of adipogenic (Fig. 2G) and osteogenic (Fig. 2H) differentiation were significantly attenuated by SERPINB2 depletion. In addition, the effects of acetylsalicylic acid on the expressions of several stemness-related transcription factors (OCT4, NANOG, and SOX2) were also abolished by its knockdown (Fig. 2I). These results indicate that SERPINB2 may act as a functional regulator to mediate the acetylsalicylic acid-induced inhibition of various endometrial stem cell functions related to regenerative capacity.

### The suppressive effects of acetylsalicylic acid on the regenerative capacity of endometrial stem cells of are mediated through the Akt and/or ERK1/2 pathways

**Fig. 3:**
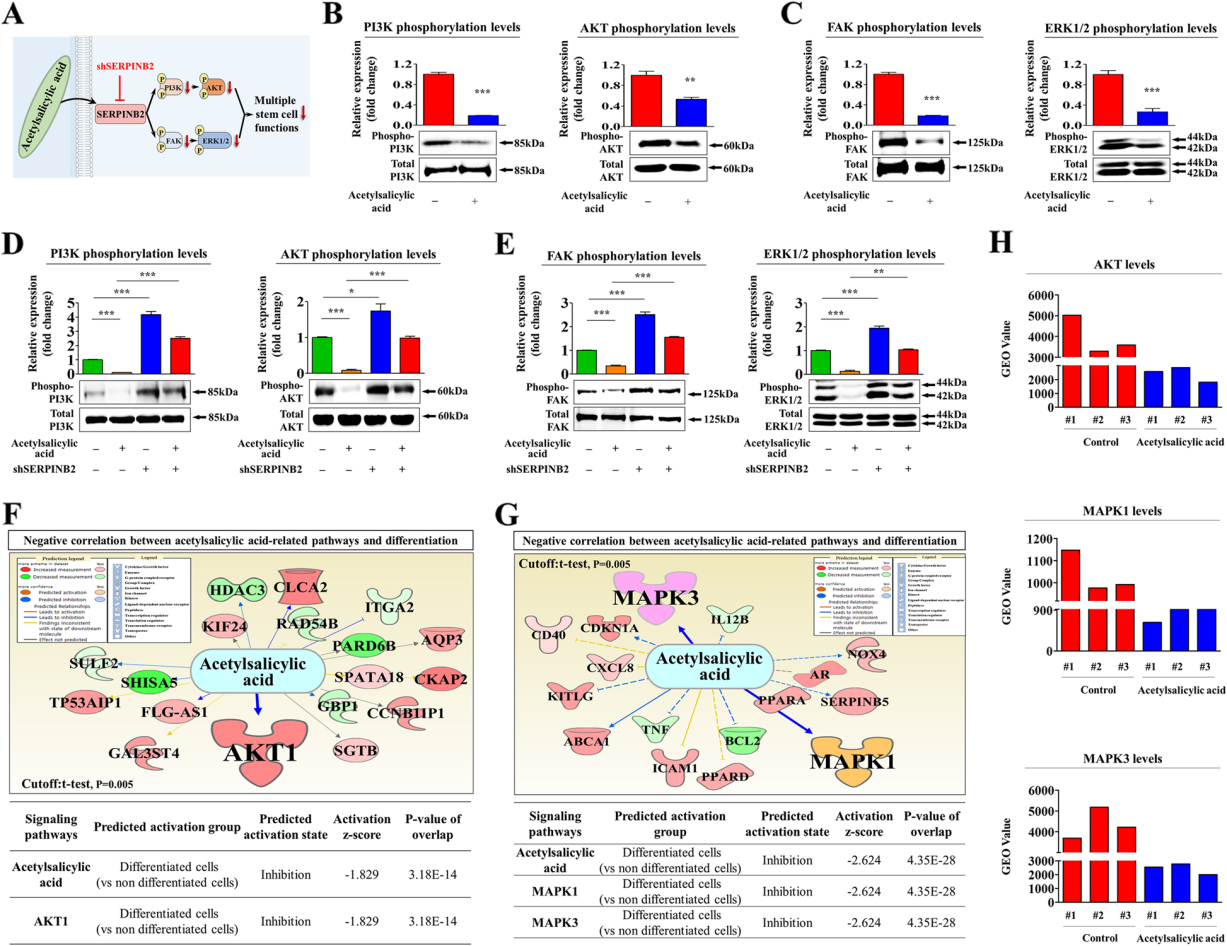
The attenuating effect of SERPINB2 knockdown on the acetylsalicylic acid-induced suppression of the Akt or ERK1/2 signaling cascade. A schematic representation of the role of SERPINB2 as an upstream regulator of the acetylsalicylic acid-induced PI3K/Akt and FAK/ERK1/2 signaling cascades is shown (**A**). Endometrial stem cells were treated with or without acetylsalicylic acid (2.5 mM) for 15 min, and the subsequent changes in the phosphorylation (activation) levels of signaling molecules (i.e., Akt, PI3K, FAK, and ERK1/2) were assessed by western blotting (**B**-**C**). Endometrial stem cells were treated with acetylsalicylic acid (2.5 mM) alone or concomitantly transfected with a specific shRNA targeting SERPINB2; the subsequent changes in the phosphorylation states of these signaling molecules were evaluated by western blotting (**D**-**E**). The activation states (either activated or inactivated) of various Akt1 (GSE100752) (**F**) or MAPK1/3 (ERK1/3) (GSE129144/GSE76381) (**G**)-associated genes/ transcription factors in proliferating and nonproliferating cells were analyzed using the ingenuity pathway analysis (IPA) software. The GEO metadata were also analyzed to further investigate the contributions of the Akt and MAPK1/3 (ERK1/3) signaling pathways to various acetylsalicylic acid treatment conditions (**H**). β-actin was used as an internal control to normalize protein expression. All experiments were performed in triplicates. Significant differences are presented. *p < 0.05, **p < 0.005, and ***p < 0.001 (One-way ANOVA)

**Fig. 4:**
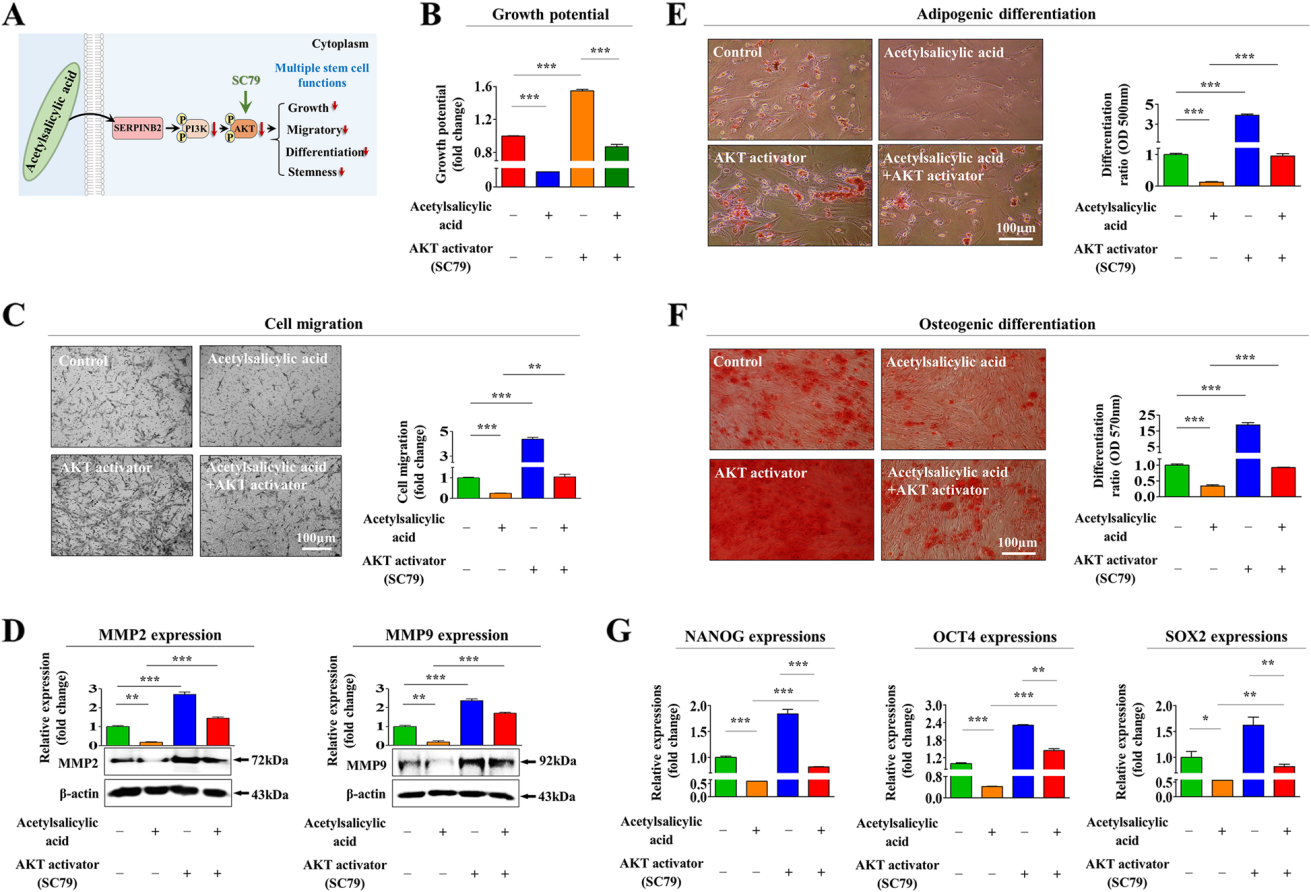
Activation of the Akt signaling cascade alleviates the acetylsalicylic acid-induced suppression of various endometrial stem cell functions related to regenerative capacity. A schematic representation of the regulatory role of the PI3K/Akt signaling cascade in mediating the effects of acetylsalicylic acid is shown (**A**). Endometrial stem cells were pretreated with the specific Akt activator SC79 (10 µM) for 1 h and then treated with acetylsalicylic acid (2.5 mM) for 48 h, and the subsequent effects of acetylsalicylic acid on self-renewal capacity were assessed using MTT assays. The stem cell proliferation rates (%) were assessed by representing the viability of the acetylsalicylic acid-treated cells as a percentage of the viability of the vehicle-treated cells (**B**). The effect of the Akt activator on the acetylsalicylic acid-induced change in migration potential was evaluated by in vitro Transwell assays **(C)** and western blotting with antibodies against MMP-2 and MMP-9 (**D**). Endometrial stem cells were pretreated with 10 µM SC79 for 1 h and then treated with 2.5 mM acetylsalicylic acid for 48, and the subsequent changes in adipogenic (**E**) and osteogenic (**F**) differentiation were analyzed by oil red O and alizarin red staining, respectively. The cytoplasmic calcium concentration and lipid droplet (LD) formation within differentiated cells were assessed by measuring the absorbance values of the solubilized cells at wavelengths of 500 nm and 570 nm, respectively. The effect of 10 µM SC79 on the acetylsalicylic acid-induced inhibition of the pluripotency-associated transcription factors NANOG, OCT4, and SOX2 was measured by real-time PCR (**G**). β-actin was used as an internal control to normalize protein expression. PPIA was used as a reference gene to normalize gene expression. All experiments were performed in triplicates. Significant differences are presented. *p < 0.05, **p < 0.005, and ***p < 0.001 (One-way ANOVA)

**Fig. 5:**
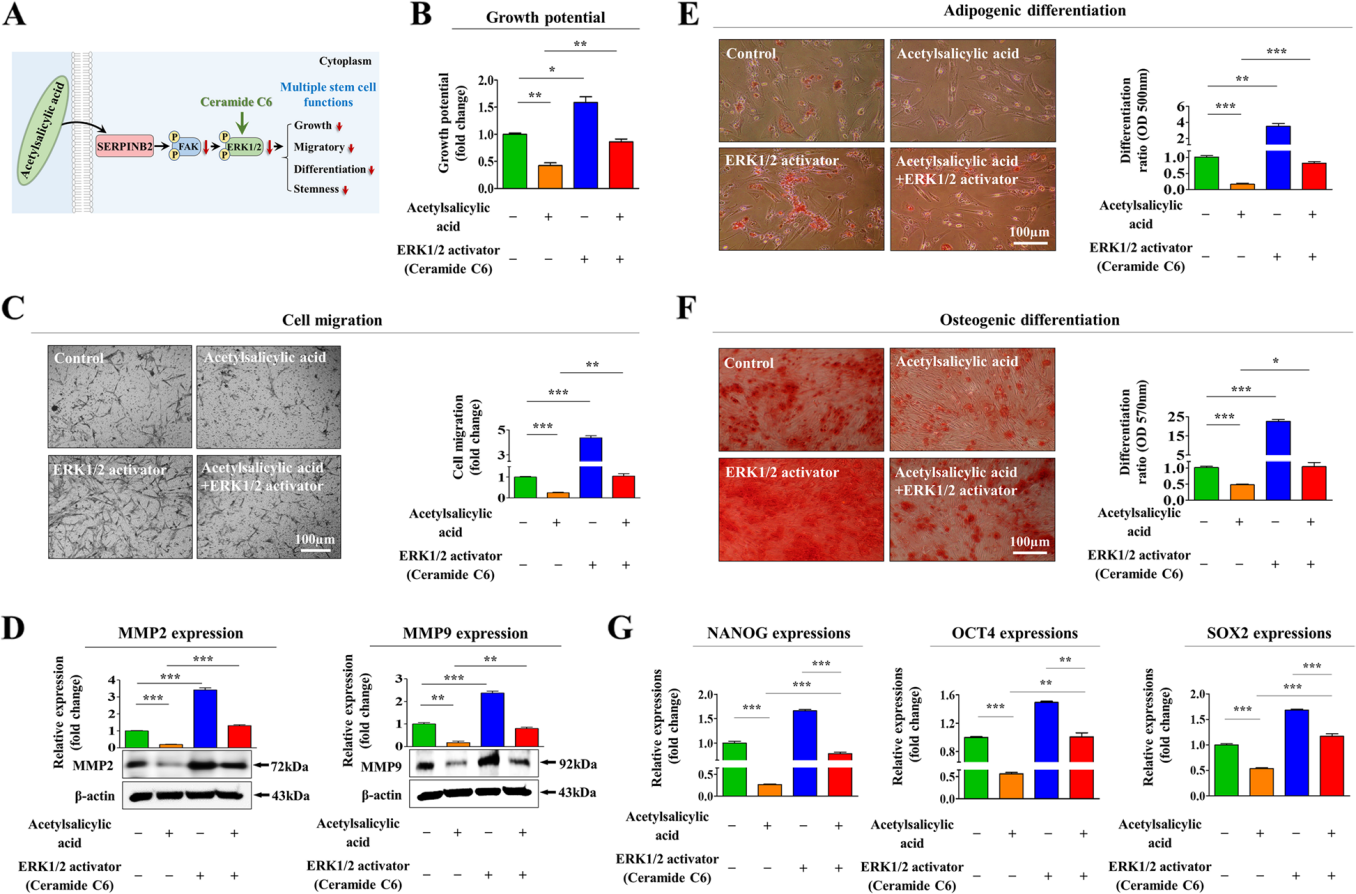
Activation of the ERK1/2 signaling cascade alleviates the acetylsalicylic acid-induced suppression of various endometrial stem cell functions related to regenerative capacity. A schematic representation of the regulatory role of the FAK/ERK1/2 signaling cascade in mediating the effects of acetylsalicylic acid is shown (**A**). Endometrial stem cells were pretreated with the specific ERK1/2 activator ceramide C6 (10 µM) for 1 h and then treated with acetylsalicylic acid (2.5 mM) for 48 h, and the subsequent effects of acetylsalicylic acid on self-renewal capacity were assessed by MTT assays. The stem cell proliferation rates (%) were assessed by representing the viability of the acetylsalicylic acid-treated cells as a percentage of the viability of the vehicle-treated cells (**B**). The effect of the ERK1/2 activator on the acetylsalicylic acid-induced changes in migratory capacity was evaluated by in vitro Transwell assays (**C**) and western blotting with antibodies against MMP-2 and MMP-9 (**D**). Endometrial stem cells were pretreated with 10 µM ERK1/2 activator ceramide C6 for 1 h and then treated with 2.5 mM treatment for 48, and the subsequent effects of acetylsalicylic acid on adipogenic (**E**) and osteogenic (**F**) differentiation were evaluated by oil red O and alizarin red staining. The cytoplasmic calcium concentration and lipid droplet (LD) formation within differentiated cells were assessed by measuring the absorbance values of the solubilized cells at wavelengths of 500 nm and 570 nm, respectively. The effect of 10 µM ceramide C6 on the acetylsalicylic acid -induced inhibition of the pluripotency-associated transcription factors NANOG, OCT4, and SOX2 was analyzed by real-time PCR (**G**). β-actin was used as an internal control to normalize protein expression. PPIA was used as a reference gene to normalize gene expression. All experiments were performed in triplicates. Significant differences are presented. *p < 0.05, **p < 0.005, and ***p < 0.001 (One-way ANOVA)

We then investigated possible molecular mechanisms underlying the acetylsalicylic acid-mediated inhibitory effects on the regenerative capacity of endometrial stem cells by assessing the effects of acetylsalicylic acid on the Akt and ERK1/2 pathways, which have previously been implicated in controlling the self-renewal [25], migratory capacities [26], and pluripotency/stemness [27] of various stem cell types (Fig. 3A). We thus analyzed whether the Akt (Fig. 3B) or ERK1/2 (Fig. 3C) signaling were suppressed by acetylsalicylic acid using western blotting. We also analyzed signaling activities of PI3K/AKT and ERK1/2 pathways following treatment with acetylsalicylic acid at different concentrations (ranging from 0.1 mM to 5 mM). As a result, we observed a significant, dose-dependent decrease in the phosphorylation levels of the PI3K/AKT (Suppl. Figure 3A and B) and ERK1/2 (Suppl. Figure 3C) pathways by acetylsalicylic acid treatment. We then assessed the effect of SERPINB2 knockdown on the acetylsalicylic acid-induced inhibition of the Akt or ERK1/2 signaling. Importantly, the inhibitory effects of acetylsalicylic acid on the Akt (Fig. 3D) or ERK1/2 (Fig. 3E) signaling were significantly abolished by SERPINB2 knockdown. These results indicate that SERPINB2 act as a potent regulator of the acetylsalicylic acid-induced Akt or ERK1/2 signaling cascades in endometrial stem cells. In addition, we evaluated the activation status of various Akt- or ERK1/2-related factors and their signaling networks to further analysis whether the activation of acetylsalicylic acid-associated signaling is negatively correlated with the Akt or ERK1/2 pathway using ingenuity pathway analysis (IPA). The expression of the negative regulators of several acetylsalicylic acid-related genes, including Akt1, CLCA2, and KIF24, were reduced in highly differentiated cells (Fig. 3F). In addition, the expression of the negative regulators of various acetylsalicylic acid-related genes, including MAPK1, MAPK2, and CDKN1A, were reduced in highly differentiated cells (Fig. 3G). Consistent with these results, the Gene Expression Omnibus (GEO) database indicated that the expressions of Akt, MAPK1, and MAPK3 are clearly reduced in various acetylsalicylic acid treatment conditions (Fig. 3H). In addition, we evaluated the activation of the Akt (Fig. 4A) or the ERK1/2 (Fig. 5A) signaling pathways on endometrial stem cell functions related to regenerative capacity, such as proliferative, migratory, stemness, and multilineage differentiation capacities, with or without acetylsalicylic acid treatment. Indeed, the acetylsalicylic acid-induced inhibitory effects on endometrial stem cell growth were clearly diminished by pretreatment with Akt (Fig. 4B) or ERK activations (Fig. 5B) with their specific activators. Akt activator SC79 (Fig. 4C-D) or ERK activator ceramide C6 (Fig. 5C-D) pretreatment significantly abolished the acetylsalicylic acid-meditated inhibitory effects on the migratory capacity and MMP-2/9 expression. The inhibitory effects on adipogenic and osteogenic differentiation were also clearly abolished by Akt (Fig. 4E-F) or ERK (Fig. 5E-F) activations. In addition, the effects of acetylsalicylic acid on the expression of stemness-associated genes, such as OCT4, NANOG, and SOX2, were also markedly attenuated by Akt (Fig. 4G) or ERK (Fig. 5G) activations. These findings highlight the potential significance of Akt and/or ERK1/2 signaling pathways in regulating various regenerative capacity associated endometrial stem cell functions through SERPINB2 signaling cascades.

### Correlation analysis of acetylsalicylic acid-induced changes in the expression of multiple growth factors and various acetylsalicylic acid treatment conditions

**Fig. 6:**
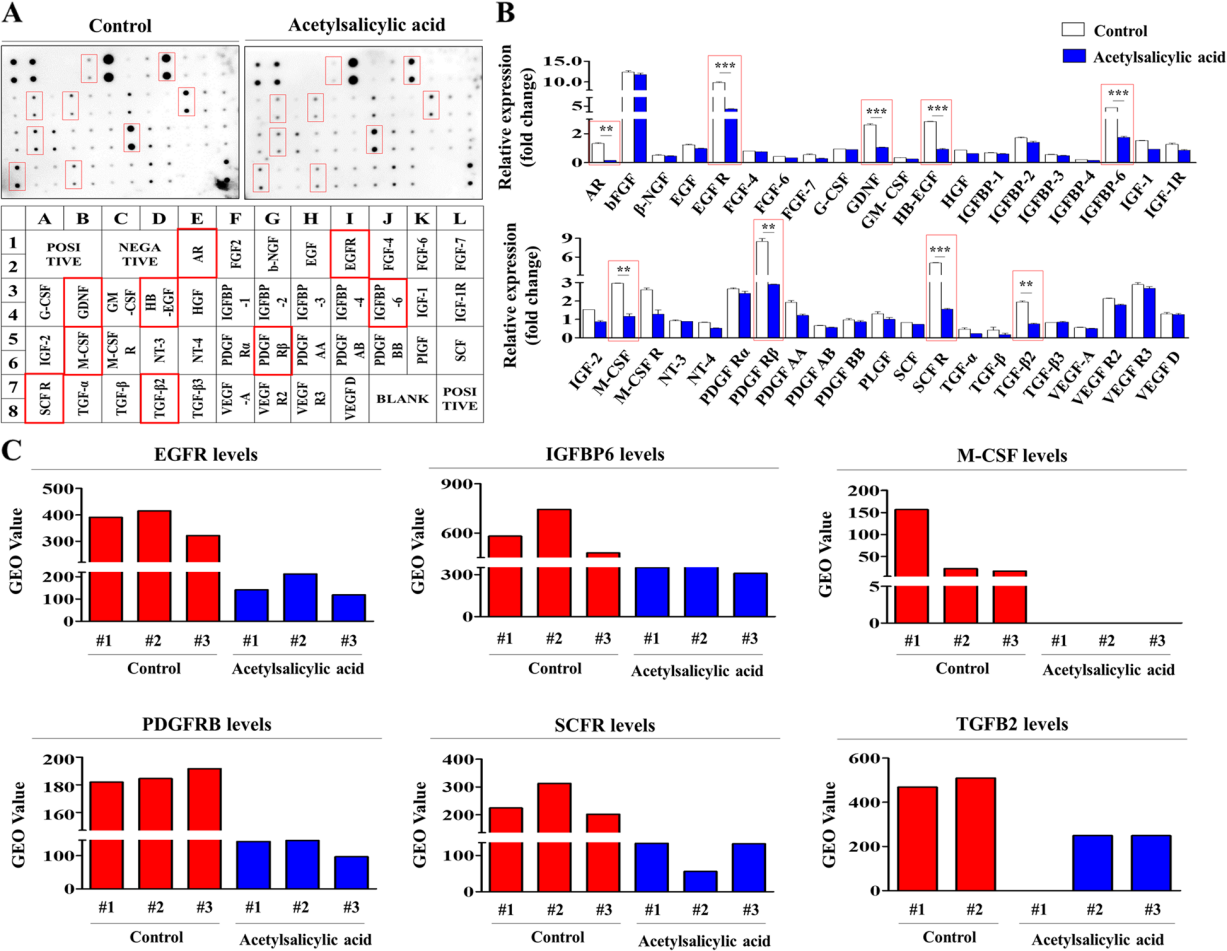
Acetylsalicylic acid treatment reduces the secretion of multiple cytokines or growth factors related to the regeneration-associated gene network in vitro. The human growth factor/cytokine antibody array was analyzed using acetylsalicylic acid-treated or nontreated protein samples. The nitrocellulose membrane was spotted with specific antibodies for 40 different cytokines, growth factors, and receptors. Nine growth factors (AR, EGFR, GDNF, HB-EGF, IGFBP-6, M-CSF, PDGFRB, SCFR, and TGFß2) were clearly reduced in the protein samples from the acetylsalicylic acid-treated cells (**A**-**B**). The GEO metadata were also analyzed to further investigate the correlations between acetylsalicylic acid treatment and the nine downregulated proteins (**C**). All experiments were performed in triplicates. Significant differences are presented. *p < 0.05, **p < 0.005, and ***p < 0.001 (One-way ANOVA)

To investigate whether the suppressive effects of acetylsalicylic acid on various endometrial stem cell functions can be affected by the release of certain cytokines or growth factors, we performed growth factor antibody arrays using cells with or without acetylsalicylic acid treatment. Our analysis revealed significant alterations in the secretions of 40 proteins in acetylsalicylic acid-treated endometrial stem cells. Although other growth factors were shown to have only slight changes in their expression, the relative levels of nine prominent growth factors, namely, androgen receptor (AR); epidermal growth factor receptor (EGFR); glial cell-derived neurotrophic factor (GDNF); insulin-like growth factor-binding protein 6 (IGFBP-6); heparin-binding EGF-like growth factor (HB-EGF); macrophage colony-stimulating factor (M-CSF); platelet-derived growth factor receptor, beta polypeptide (PDGFRB); stem cell factor receptor (SCFR); and transforming growth factor 2 (TGFB2) were significantly reduced by acetylsalicylic acid treatment (Fig. 6A-B). These results indicate that these growth factors are probably responsible for the acetylsalicylic acid-induced suppression of the Akt or ERK1/2 signaling and its subsequent inhibition of regenerative capacities of endometrial stem cells. Furthermore, the GEO dataset also revealed that the expressions of the nine prominent growth factors detected are decreased in response to various acetylsalicylic acid treatment (Fig. 6C). We analyzed various signaling networks using the IPA software to further assess whether the nine growth factors detected are related to the signaling pathways controlling proliferative capacity. Positive regulators of EGFR, such as EGF, EGFR, and MAPK3, are activated in highly proliferating cells (Suppl. Figure 4A). Positive regulators of IGFBP6, such as FGF21 and PDGFBB, are activated in highly proliferating cells (Suppl. Figure 4B). Positive regulators of M-CSF2, such as FGFR2 and MAPK1, are activated in highly proliferating cells (Suppl. Figure 4C). Positive regulators of DGFRB, such as JUN and TGFB1, are activated in highly proliferating cells (Suppl. Figure 4D). Positive regulators of SCFR, such as HGF and VEGFA, are activated in highly proliferating cells (Suppl. Figure 4E). Positive regulators of TGFB2, such as AREG and FOS, are activated in highly proliferating cells (Suppl. Figure 4F). Taken together, these results indicate that these secreted proteins may be potent upstream regulators that affect the ERK1/2 or Akt signaling to mediate the effects of acetylsalicylic acid.

### Acetylsalicylic acid treatment inhibits various functions related to the regenerative capacities of tissue-resident endometrial stem cells in vivo

**Fig. 7:**
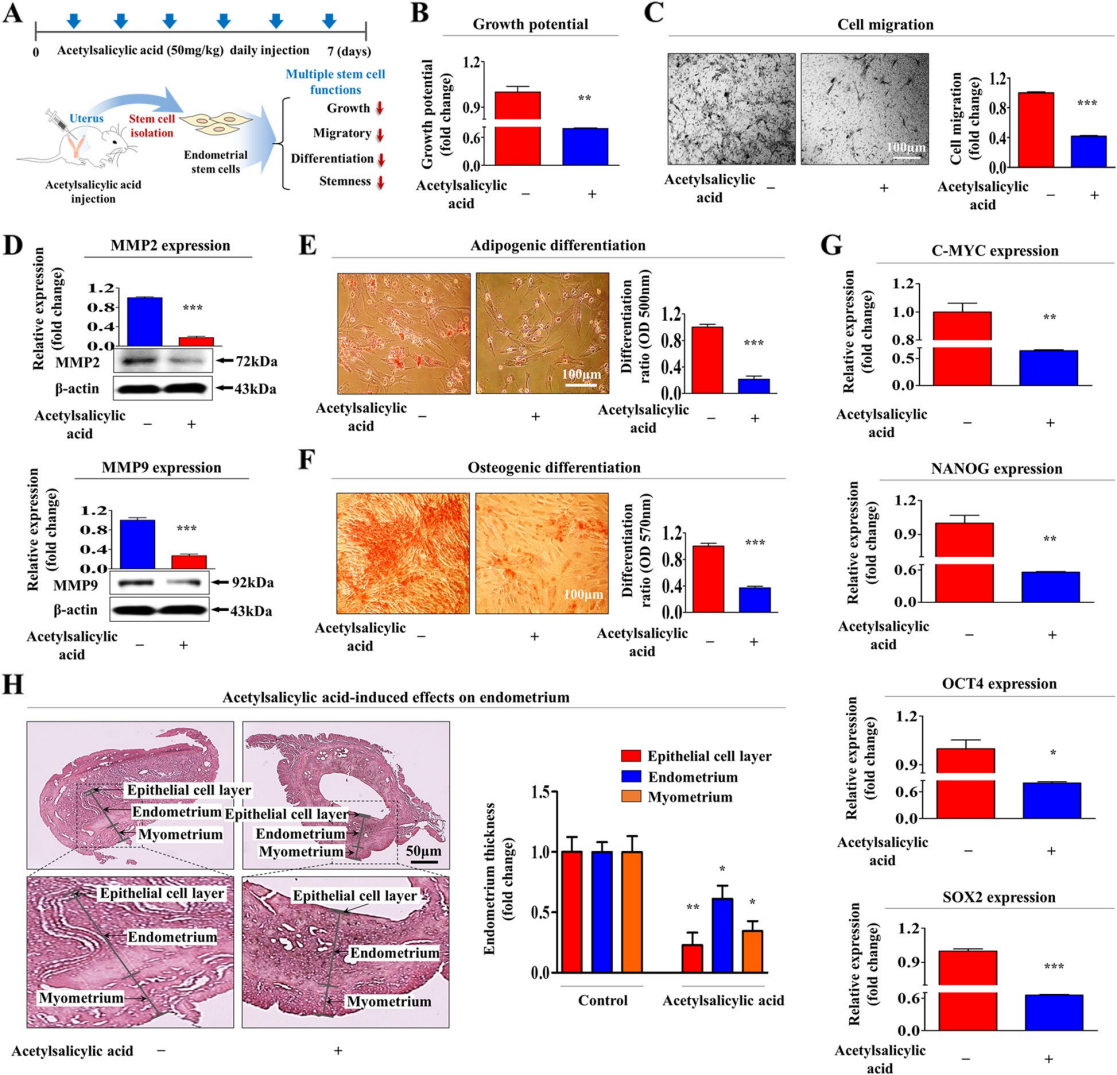
Acetylsalicylic acid treatment markedly inhibits multiple beneficial functions of endometrial stem cells in vivo. A schematic representation of the experimental procedure as described in the Materials and Methods section is shown (**A**). The mice were treated intravenously with acetylsalicylic acid (50 mg/kg daily for 7 consecutive days), and the endometrial stem cells were isolated from the mouse uterine endometrium using our primary culture technique. The isolated endometrial stem cells were cultured in vitro either under continuous exposure to acetylsalicylic acid (2.5 mM) or in non-acetylsalicylic acid culture conditions to mimic the physiological environment of acetylsalicylic acid exposure in vivo. The subsequent inhibition of the self-renewal capacity of mouse endometrial stem cells was analyzed by MTT assays. The stem cell proliferation rates (%) were assessed by representing the viability of the acetylsalicylic acid-treated cells as a percentage of the viability of the vehicle-treated cells (**B**). The acetylsalicylic acid -induced inhibition of migratory potential in vivo was then analyzed by Transwell migration/invasion assays (**C**) and western blotting with antibodies against MMP-2 and MMP-9 (**D**). The acetylsalicylic acid-induced inhibition of adipogenic (**E**) and osteogenic (**F**) differentiation in vivo were analyzed by oil red O and alizarin red staining, respectively. The cytoplasmic calcium concentration and lipid droplet (LD) formation within differentiated cells were assessed by measuring the absorbance values of the solubilized cells at wavelengths of 500 nm and 570 nm, respectively. The acetylsalicylic acid-induced inhibition of the expression of various pluripotency/stemness markers (NANOG, OCT4, and SOX2) in vivo was evaluated using real-time PCR (**G**). Mice from each group were sacrificed by cervical dislocation. Uterine endometrial tissue samples from acetylsalicylic acid-treated mice were collected and fixed in 10% formalin for hematoxylin and eosin (H&E) staining. The histological evaluation revealed that the functional layer of the mouse endometrium was significantly decreased by acetylsalicylic acid treatment in vivo (**H**). β-actin was used as an internal control to normalize protein expression. All experiments were performed in triplicates. HPRT was used as a reference gene to normalize gene expression. Significant differences are presented. *p < 0.05, **p < 0.005, and ***p < 0.001 (One-way ANOVA)

Our in vitro data indicate that acetylsalicylic acid treatment may also suppress regenerative capacities associated functions of endometrial stem cells in vivo. Therefore, acetylsalicylic acid (50 mg/kg) was administered daily to mice through intraperitoneal (i.p.) injection for 7 days. Tissue-resident endometrial stem cells were then isolated from the mouse uterine tissue and cultured (Fig. 7A). Similar to our in vitro data, the results showed that acetylsalicylic acid treatment markedly reduced the self-renewal ability of endometrial stem cells (Fig. 7B). Transwell invasion assays also revealed the suppressive effects of acetylsalicylic acid treatment on the migration potential of endometrial stem cells in vivo (Fig. 7C)*.* The acetylsalicylic acid-induced inhibition of endometrial stem cell migration was further assessed by western blot analysis using MMP-2/9 antibodies (Fig. 7D). In addition, acetylsalicylic acid treatment significantly decreased their adipogenic (Fig. 7E) and osteogenic (Fig. 7F) differentiation capacity in vivo. Real-time PCR results revealed that the levels of the stemness-associated transcription factors OCT4, NANOG, C-MYC, and SOX2 were markedly decreased by acetylsalicylic acid treatment (Fig. 7G). Importantly, we also investigated whether acetylsalicylic acid treatment could affect the regenerative capacity of the endometrium, which largely rely on the local endometrial stem cells in vivo. Our histological analysis showed that the thickness of the endometrial layer was clearly reduced by consecutive acetylsalicylic acid treatment (Fig. 7H). We further investigated whether acetylsalicylic acid treatment also suppresses regenerative capacities of other relevant types of stem cells, such as adipose tissue-derived stem cells (Suppl. Figure 5A) and bone marrow-derived stem cells (Suppl. Figure 6A) in vivo. Similar to endometrial stem cell results, acetylsalicylic acid treatment clearly reduced the self-renewal (Suppl. Figure 5B and 6B), migratory (Suppl. Figure 5C/D and 6C/D), and differentiation capacities (Suppl. Figure 5E/F and 6E/F) of these tissue-resident stem cells. Furthermore, the expressions of the stemness-associated genes NANOG, OCT4, C-MYC, and SOX2 were significantly decreased by acetylsalicylic acid treatment in both adipose tissue stem cells (Suppl. Figure 5G) and bone marrow stem cells (Suppl. Figure 6G) in vivo. Taken together, these findings suggest that consecutive acetylsalicylic acid treatment negatively affects the regenerative capacity of the endometrial stem cells by reducing their self-renewal, migratory, and differentiation potentials.

## Discussion

Intensive investigation of critical factors and cytokines that influence the quality of endometrial stem cells could lead to a better understanding of female infertility or repeated abortions that were previously unexplained. While there are various factors that can influence the functions of endometrial stem cells, the effects of acetylsalicylic acid have recently garnered attention due to its emerging regulatory functions in different disease conditions, such as cancers [28], gastric ulcers [29], hypertension [30], inflammation [31], and infertility [32]. Acetylsalicylic acid (also known as Aspirin) is one of the most potent and widely used medicines ever discovered [33]. Since its discovery more than 100 years ago [34], acetylsalicylic acid has been used clinically for its anti-inflammatory, anti-pyretic, and anti-nociceptive effects [35]. The therapeutic mechanisms of acetylsalicylic acid have only been revealed in recent decades [36], beginning with its antiprostaglandin effects [37]. Interestingly, the potential impact of acetylsalicylic acid on endometrial receptivity and subsequent pregnancy rates is still remains a topic of debate due to conflicting data: While some studies have shown positive effects of acetylsalicylic acid [38, 39], others have found that it has no effect [40]. Previous studies have examined the direct effects in endometrial cancer cell models in vitro [41]. However, there is still much to investigate about the effects of acetylsalicylic acid on the various functions of normal endometrial cells and its underlying mechanisms involved. Recent research has raised new and challenging questions regarding the potential direct effect of acetylsalicylic acid on the regenerative capacities of endometrial stem cells, which play a critical role in endometrial regeneration and subsequent endometrial receptivity. In the present study, we obtained human endometrial stem cells from patients with uterine fibroids. However, we isolated these stem cells from unaffected normal tissue regions rather than uterine fibroid lesions. Therefore, we are confident that uterine fibroids do not affect the characteristics of these endometrial stem cells. Furthermore, as a result of analyzing the characteristics of isolated uterine stem cells using diverse cell surface markers (CD34, CD44, CD45, CD73, CD105, CD140b, CD146, and susD2), it becomes evident that these stem cells exhibit the typical characteristics of normal adult stem cells (Suppl. Figure 1B). Therefore, our findings suggest that uterine fibroids do not exert significant influence on the properties of isolated endometrial stem cells. In addition to expressing various surface markers, we induced the isolated endometrial stem cells to differentiate into adipocytes and osteoblasts to assess their normal differentiation abilities. As a result, we confirmed that the isolated endometrial stem cells indeed possessed the typical multilineage differentiation potential, demonstrated by their ability to differentiate into both adipocytes (Suppl. Figure 1C) and osteoblasts (Suppl. Figure 1D), with no significant impact from uterine fibroids. Previously, Cao et al. found that acetylsalicylic acid promotes the osteogenic differentiation of bone marrow mesenchymal stem cells (BM-MSCs) in vitro and in vivo, and BM-MSCs pretreated with acetylsalicylic acid have a better regenerative potential to repair calvarial bone defects in a swine model [42]. Similarly, Tang showed that acetylsalicylic acid treatment significantly improves the immunomodulatory properties of BM-MSCs via the 15d-PGJ2/PPARγ/TGF-β1 pathway and that BM-MSCs pretreated with acetylsalicylic acid significantly ameliorate disease activity in vivo [43]. In contrast to these positive outcomes, Hao et al. recently found that acetylsalicylic acid treatment significantly decreased the growth potential of BM-MSCs in a dose-dependent manner [44]. Zhan et al. also observed that acetylsalicylic acid suppressed the adipogenic differentiation of BM-MSCs by disrupting epigenetic modifications in the cells [45]. These results indicate that similar to its effects on endometrial receptivity, the effect of acetylsalicylic acid on various stem cell functions is still controversial, and the underlying mechanisms are not well understood. Furthermore, it is important to acknowledge that the dosage of acetylsalicylic acid utilized in this study exceeds clinical practice norms, with a dosage 2–3 times higher than typically administered. Moreover, the intravenous (IV) administration protocol eliminates the first-pass effect that occurs when the drug is taken orally, thereby maximizing absorption into local tissues [46]. Consequently, it is reasonable to anticipate that a substantial quantity of doses can effectively reach the cells within the local tissue. Hence, it is plausible that the elevated concentration of acetylsalicylic acid within the local tissue may induce cell death, consequently diminishing various tissue regeneration-associated functions of endometrial stem cells, including self-renewal, migratory capacity, and pluripotency/stemness.

In the present study, we found for the first time that acetylsalicylic acid treatment markedly suppressed multiple beneficial functions of endometrial stem cells, such as their proliferative, migration, and transdifferentiation capacities, both in vitro (Fig. 1A-G) and animal model (Fig. 7A-H), all of which are essential functions for endometrial receptivity [47]. Importantly, the acetylsalicylic acid-induced suppression of endometrial stem cell growth (Fig. 2C), migration (Fig. 2D-E), multilineage differentiation (Fig. 2F-G) and pluripotency (Fig. 2H) were significantly attenuated by SERPINB2 depletion using specific shRNA. Previously, it has been reported that SERPINB2 levels are significantly increased in response to various differentiation-inducing agents in various cell types, such as leukemic cells [48], mononuclear cells [49], and keratinocytes [50], suggesting that SERPINB2 may be involved in the adverse effects of acetylsalicylic acid on stem cells as a potent downstream target of acetylsalicylic acid. Consistent with this model, our previous results also revealed that enhanced SERPINB2 levels markedly decreased the proliferation and pluripotency of stem cells [51]. Cho et al. also found that stem cell senescence (aging) may enhance SERPINB2 expression, which in turn suppresses the anti-aging activity of Sonic hedgehog [21]. In addition, other studies also found that increased SERPINB2 expression suppresses self-renewal ability and is associated with elevated levels of various differentiation-related markers [52]. These findings suggest that SERPINB2 could play a crucial role in regulating the various effects of acetylsalicylic acid on endometrial stem cell functions. The diverse acetylsalicylic acid-induced effects were markedly attenuated by the activation of Akt (Fig. 4A-G) and ERK1/2 (Fig. 5A-G) signaling pathways. Moreover, we observed that the inhibitory effects of acetylsalicylic acid on the Akt and ERK1/2 signaling were markedly reduced upon SERPINB2 depletion. (Fig. 3D and E). These results indicate that the PI3K/Akt and FAK/ERK1/2 signaling pathways may be involved in the acetylsalicylic acid-induced inhibition of various endometrial stem cell functions related to regenerative capacity as potent downstream modulators of SERPINB2 signaling cascades. While acetylsalicylic acid can directly influence various tissue regeneration-related functions, such as self-renewal, migratory capacity, differentiation potential, and pluripotency/stemness of endometrial stem cells, it is also plausible that its mechanism of action involves the regulation of several growth factors pivotal for stem cell function. Indeed, treatment with acetylsalicylic acid resulted in a significant reduction in the expression of growth factors such as AR, EGFR, GDNF, IGFBP-6, HB-EGF, M-CSF, PDGFRB, SCFR, and TGFB2, all of which are known to play crucial roles in governing stem cell function (Fig. 6A-B). It is postulated that these nine growth factors, whose expression was reduced by acetylsalicylic acid, may indirectly suppress the activity of the Akt and/or ERK1/2 signaling pathways. Therefore, to substantiate this hypothesis, further investigation is required, involving the overexpression of these nine growth factors either individually or in combination, followed by acetylsalicylic acid treatment, and subsequent assessment of Akt and/or ERK1/2 signaling pathway activities.

## Conclusion

In conclusion, our study has revealed that acetylsalicylic acid exerts complex and varied effects on multiple functions of endometrial stem cells. However, the impact of these effects on endometrial receptivity and subsequent pregnancy outcomes remains unclear due to inconsistencies in previous studies. While some studies have reported beneficial effects of acetylsalicylic acid on endometrial receptivity and pregnancy outcomes, others have reported adverse effects. These inconsistencies may be explained by the differences in anatomy, genetics, and environmental factors. It is also widely recognized that animal models have limitations in accurately reflecting the physiological complexity of humans. Moreover, it remains unclear whether the effects of acetylsalicylic acid on endometrial stem cells extend to other types of endometrial constituting cells. Specifically, it is unknown whether acetylsalicylic acid exerts similar effects on endometrial stromal cells, glandular epithelial cells, and vascular endothelial cells. However, although there are still some unanswered questions, our findings may facilitate the development of more effective therapeutic strategies to increase pregnancy rates for patients with female infertility.

### Supplementary Information

**Additional file 1:** **Supplementary figure 1.** Isolation and characterization of multipotent endometrial stem cells from human uterine tissue samples. Endometrial tissue was minced into small pieces, and then the small pieces were digested with type I collagenase. Isolated human endometrial stem cells were observed under an inverted phase-contrast microscope to assess their morphological characterization (A). The isolated endometrial stem cells were analyzed using flow cytometry with various antibodies for identified stem cell markers (CD44, CD73, CD105, CD140b, CD146, and susD2) and several hematopoietic markers (CD34 and CD45) (B). Their ability to differentiate into adipocytes (C) and osteoblasts (D) was analyzed using oil red O and alizarin red S staining, respectively. The cytoplasmic calcium concentration and lipid droplet (LD) formation within differentiated cells were assessed by measuring the absorbance values of the solubilized cells at wavelengths of 500 nm and 570 nm, respectively. Significant differences are presented. *p< 0.05, **p < 0.005, and ***p < 0.001 (two-sample t-test). **Supplementary figure 2.** Knockdown efficacy of multiple shRNA constructs specifically targeting SERPINB2. Endometrial stem cells were transfected with multiple shRNA constructs #1, #2, #3, #4, or#5, which specifically target SERPINB2, or with a non-targeting shRNA control for non-specific effects (A). SERPINB2 shRNA construct #2, hereafter described as SERPINB2 shRNA, was the most effective in endometrial stem cells. The knockdown efficacy of SERPINB2 was analyzed based on mRNA (B) and protein levels (C). β-actin was used as the internal control. Significant differences are presented. *p < 0.05, **p < 0.005, and ***p< 0.001 (One-way ANOVA). **Supplementary figure 3.** The dose-dependent effects of acetylsalicylic acid on the activities of the PI3K/Akt or ERK1/2 signaling cascades in endometrial stem cells. Endometrial stem cells were treated with or without acetylsalicylic acid at different concentrations (ranging from 0.1mM to 5mM) for 15 min, and the subsequent changes in the phosphorylation (activation) levels of signaling molecules (i.e., Akt, PI3K, and ERK1/2) were assessed by western blotting (A-C). β-actin was used as an internal control to normalize protein expression. All experiments were performed in triplicates. Significant differences are presented. *p < 0.05, **p < 0.005, and ***p < 0.001 (One-way ANOVA). **Supplementary figure 4.** The signaling networks of the various acetylsalicylic acid -induced prominent factors are positively correlated with self-renewal capacity. The differential activation status (either activated or inhibited) of various signaling pathways, such as EGFR (GSE21618), IGFBP6 (GSE47856), M-CSF (GSE45630), PDGFRB (GSE116237), SCFR (GSE46045), or TGFß2 (GSE48990)-related genes, between proliferative cells and non-proliferative cells was analyzed using IPA software (A-F). **Supplementary figure 5.** Acetylsalicylic acid treatment significantly inhibits various regenerative capacity-related functions of adipose tissue-derived stem cells *in vivo*. A schematic representation of the experimental procedure as described in the Materials and Methods section is shown (A). The mice were treated intravenously with acetylsalicylic acid (50 mg/kg daily for 7 consecutive days), and tissue resident stem cells were isolated from mouse adipose tissues using our primary culture technique. The isolated adipose tissue-derived stem cells were cultured *in vitro *either under continuous exposure to acetylsalicylic acid (2.5 mM) or in non-acetylsalicylic acid culture conditions to mimic the physiological environment of acetylsalicylic acid exposure *in vivo*. The subsequent inhibition of the self-renewal capacity of mouse adipose tissue-derived stem cells was analyzed by MTT assays. The stem cell proliferation rates (%) were assessed by representing the viability of the acetylsalicylic acid-treated cells as a percentage of the viability of the vehicle-treated cells (B). The acetylsalicylic acid-induced inhibition of migratory potential *in vivo *was then analyzed by Transwell migration/invasion assays (C) and western blotting with antibodies against MMP-2 and MMP-9 (D). The acetylsalicylic acid-induced inhibition of adipogenic (E) and osteogenic (F) differentiation *in vivo *were analyzed by oil red O and alizarin red staining, respectively. The cytoplasmic calcium concentration and lipid droplet (LD) formation within differentiated cells were assessed by measuring the absorbance values of the solubilized cells at wavelengths of 500 nm and 570 nm, respectively. The acetylsalicylic acid-induced inhibition of the expression of various pluripotency/stemness markers (C-MYC, NANOG, OCT4, and SOX2) *in vivo *was evaluated using real-time PCR (G). β-actin was used as the internal control. HPRT was used as a housekeeping gene for real-time PCR analysis. Significant differences are presented. *p< 0.05, **p < 0.005, and ***p < 0.001 (One-way ANOVA). **Supplementary figure 6.** Acetylsalicylic acid treatment significantly inhibits various regenerative capacity-related functions of bone marrow stem cells *in vivo*. A schematic representation of the experimental procedure as described in the Materials and Methods section is shown (A). The mice were treated intravenously with acetylsalicylic acid (50 mg/kg daily for 7 consecutive days), and tissue resident stem cells were isolated from mouse bone marrow using our primary culture technique. The isolated bone marrow stem cells were cultured *in vitro *either under continuous exposure to acetylsalicylic acid (2.5 mM) or in non-acetylsalicylic acid culture conditions to mimic the physiological environment of acetylsalicylic acid exposure *in vivo*. The subsequent inhibition of the self-renewal capacity of mouse bone marrow stem cells stem cells was analyzed by MTT assays. The stem cell proliferation rates (%) were assessed by representing the viability of the acetylsalicylic acid-treated cells as a percentage of the viability of the vehicle-treated cells (B). The acetylsalicylic acid-induced inhibition of migratory potential *in vivo *was then analyzed by Transwell migration/invasion assays (C) and western blotting with antibodies against MMP-2 and MMP-9 (D). The acetylsalicylic acid-induced inhibition of adipogenic (E) and osteogenic (F) differentiation *in vivo *were analyzed by oil red O and alizarin red staining, respectively. The cytoplasmic calcium concentration and lipid droplet (LD) formation within differentiated cells were assessed by measuring the absorbance values of the solubilized cells at wavelengths of 500 nm and 570 nm, respectively. The acetylsalicylic acid-induced inhibition of the expression of various pluripotency/stemness markers (C-MYC, NANOG, OCT4, and SOX2) *in vivo *was evaluated using real-time PCR (G). β-actin was used as the internal control. HPRT was used as a housekeeping gene for real-time PCR analysis. Significant differences are presented. *p < 0.05, **p < 0.005, and ***p< 0.001 (One-way ANOVA).

## Data Availability

The data that support the findings of this study and materials are available from the corresponding author upon reasonable request.

## Abbreviations

AR: Androgen receptor
AREG: Amphiregulin
BM-MSCs: Bone marrow-derived mesenchymal stromal cells
CDKN1A: Cyclin Dependent Kinase Inhibitor 1A
CLCA2: Chloride Channel Accessory 2
COX-1 and 2: Cyclooxygenases-1 and 2.
ECM: Extracellular matrix
EGFR: Epidermal growth factor receptor
ERK1/2: Extracellular signal-regulated kinase 1/2
GEO: Gene expression omnibus
HB-EGF: Heparin-binding EGF-like growth factor
HGF: Hepatocyte growth factor
KIF24: Kinesin family member 24
FGF21: Fibroblast growth factor 21
FGFR2: Fibroblast growth factor receptor 2
IVF: in vitro Fertilization
IGFBP-6: Insulin-like growth factor-binding protein 6
M-CSF: Macrophage colony-stimulating factor
MAPK: Mitogen-activated protein kinase
MMP-2/9: Metalloproteinases-2/9
PDGFRB: Platelet-derived growth factor receptor, beta polypeptide
RCTs: Randomized controlled trials
PGJ2: (Prostaglandin J2)
PPARγ: Peroxisome proliferator-activated receptor gamma
RPL: Recurrent early pregnancy loss
TGFB2: Transforming growth factor 2
SCFR: Stem cell factor receptor
VEGFA: Vascular endothelial growth factor A
