# Supplementary material for: Unveiling the potential effects of acetylsalicylic acid: insights into regeneration in endometrial stem cells
Source: Cell Commun Signal. 2023 Nov 10;21:323. doi: 10.1186/s12964-023-01339-2 (PMC10638813; doi:10.1186/s12964-023-01339-2)
Supplement: Supplementary file 2 — Additional file 1: Supplementary figure 1. Isolation and characterization of multipotent endometrial stem cells from human uterine tissue samples. Endometrial tissue was minced into small pieces, and then the small pieces were digested with type I collagenase. Isolated human endometrial stem cells were observed under an inverted phase-contrast microscope to assess their morphological characterization (A). The isolated endometrial stem cells were analyzed using flow cytometry with various antibodies for identified stem cell markers (CD44, CD73, CD105, CD140b, CD146, and susD2) and several hematopoietic markers (CD34 and CD45) (B). Their ability to differentiate into adipocytes (C) and osteoblasts (D) was analyzed using oil red O and alizarin red S staining, respectively. The cytoplasmic calcium concentration and lipid droplet (LD) formation within differentiated cells were assessed by measuring the absorbance values of the solubilized cells at wavelengths of 500 nm and 570 nm, respectively. Significant differences are presented. *p< 0.05, **p < 0.005, and ***p < 0.001 (two-sample t-test). Supplementary figure 2. Knockdown efficacy of multiple shRNA constructs specifically targeting SERPINB2. Endometrial stem cells were transfected with multiple shRNA constructs #1, #2, #3, #4, or#5, which specifically target SERPINB2, or with a non-targeting shRNA control for non-specific effects (A). SERPINB2 shRNA construct #2, hereafter described as SERPINB2 shRNA, was the most effective in endometrial stem cells. The knockdown efficacy of SERPINB2 was analyzed based on mRNA (B) and protein levels (C). β-actin was used as the internal control. Significant differences are presented. *p < 0.05, **p < 0.005, and ***p< 0.001 (One-way ANOVA). Supplementary figure 3. The dose-dependent effects of acetylsalicylic acid on the activities of the PI3K/Akt or ERK1/2 signaling cascades in endometrial stem cells. Endometrial stem cells were treated with or without acetylsalicylic acid at differ [file 12964_2023_1339_MOESM1_ESM.pdf]

Supplementary figure legends

Supplementary figure 1

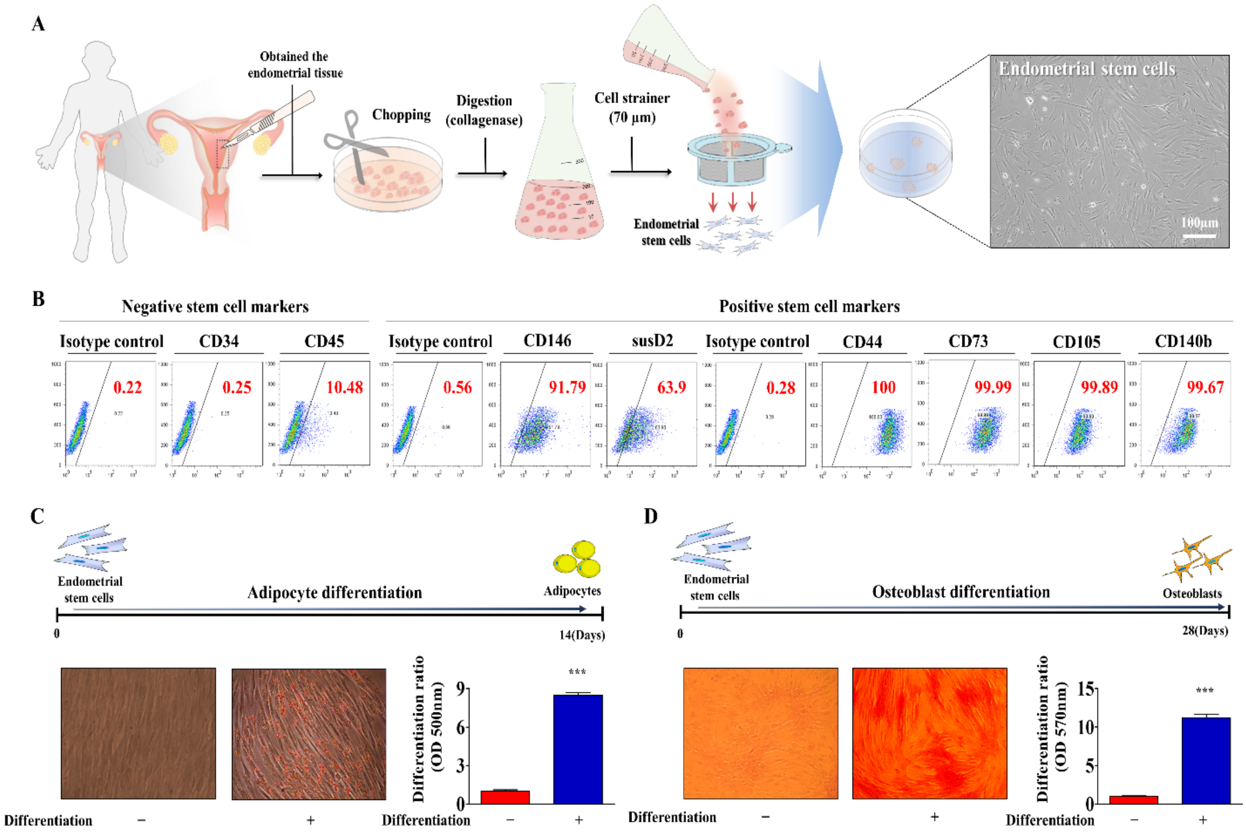

Supplementary figure 1. Isolation and characterization of multipotent endometrial stem cells from human uterine tissue samples. Endometrial tissue was minced into small pieces, and then the small pieces were digested with type I collagenase. Isolated human endometrial stem cells were observed under an inverted phase-contrast microscope to assess their morphological characterization **(A)**. The isolated endometrial stem cells were analyzed using flow cytometry with various antibodies for identified stem cell markers (CD44, CD73, CD105, CD140b, CD146, and susD2) and several hematopoietic markers (CD34 and CD45) **(B)**. Their ability to differentiate into adipocytes **(C)** and osteoblasts **(D)** was analyzed using oil red O and alizarin red S staining, respectively. The cytoplasmic calcium concentration and lipid droplet (LD) formation within differentiated cells were assessed by measuring the absorbance values of the solubilized cells at wavelengths of 500 nm and 570 nm, respectively. Significant differences are presented. *$p < 0.05$, **$p < 0.005$, and ***$p < 0.001$ (two-sample t-test).

Supplementary figure 2

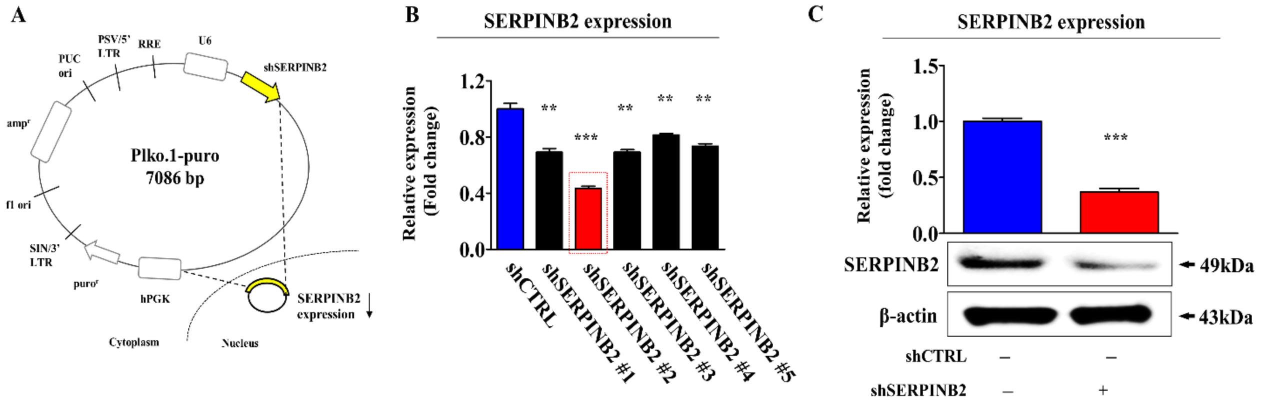

Supplementary figure 2. Knockdown efficacy of multiple shRNA constructs specifically targeting SERPINB2. Endometrial stem cells were transfected with multiple shRNA constructs #1, #2, #3, #4, or #5, which specifically target SERPINB2, or with a non-targeting shRNA control for non-specific effects **(A)**. SERPINB2 shRNA construct #2, hereafter described as SERPINB2 shRNA, was the most effective in endometrial stem cells. The knockdown efficacy of SERPINB2 was analyzed based on mRNA **(B)** and protein levels **(C)**. β-actin was used as the internal control. Significant differences are presented. $*p < 0.05$, $**p < 0.005$, and $***p < 0.001$ (One-way ANOVA).

Supplementary figure 3

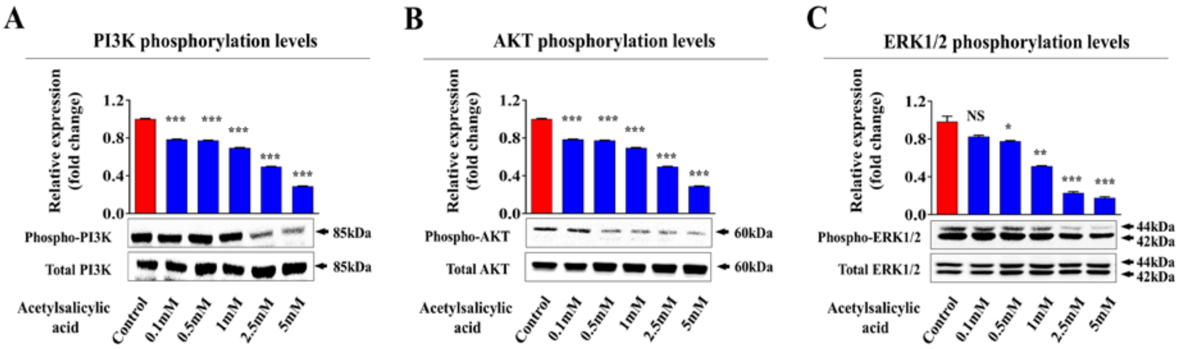

Supplementary figure 3. The dose-dependent effects of acetylsalicylic acid on the activities of the PI3K/Akt or ERK1/2 signaling cascades in endometrial stem cells. Endometrial stem cells were treated with or without acetylsalicylic acid at different concentrations (ranging from 0.1mM to 5mM) for 15 min, and the subsequent changes in the phosphorylation (activation) levels of signaling molecules (i.e., Akt, PI3K, and ERK1/2) were assessed by western blotting **(A-C)**. β-actin was used as an internal control to normalize protein expression. All experiments were performed in triplicates. Significant differences are presented. *$p < 0.05$, **$p < 0.005$, and ***$p < 0.001$ (One-way ANOVA).

Supplementary figure 4

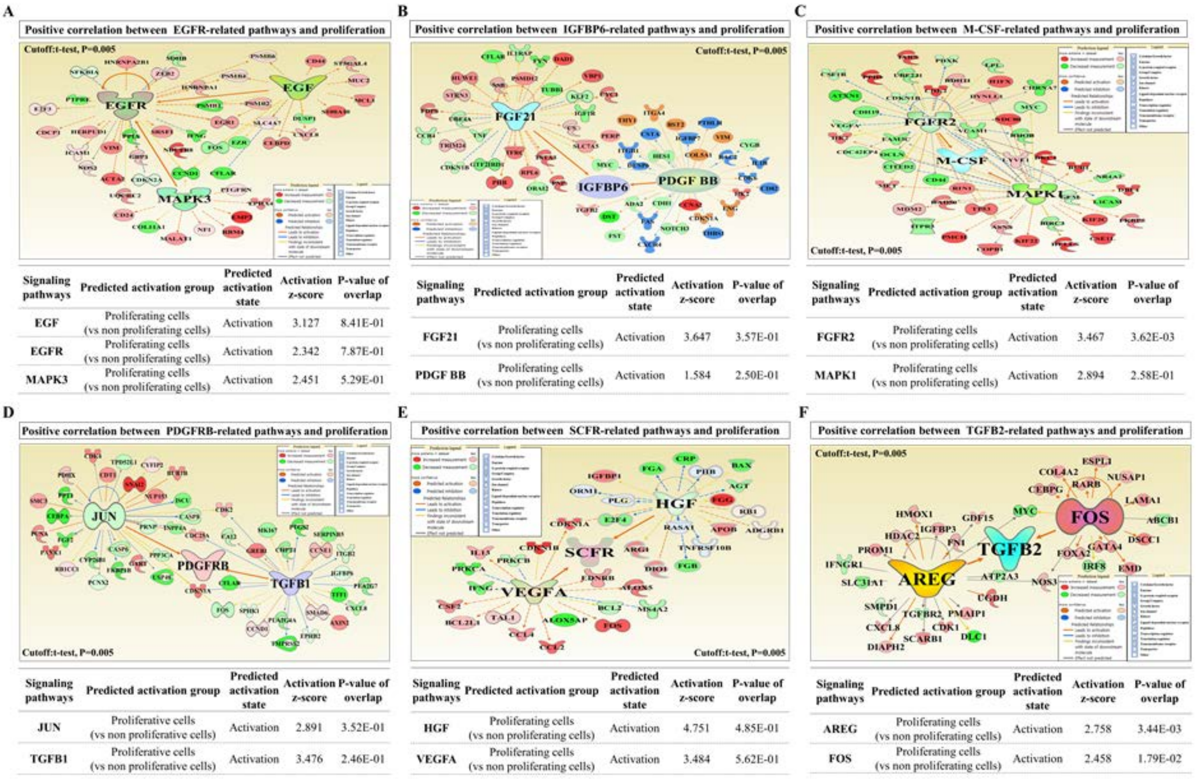

Signaling pathways	Predicted activation group	Predicted activation state	Activation z-score	P-value of overlap
EGF	Proliferating cells (vs non proliferating cells)	Activation	3.127	8.41E-01
EGFR	Proliferating cells (vs non proliferating cells)	Activation	2.342	7.87E-01
MAPK3	Proliferating cells (vs non proliferating cells)	Activation	2.451	5.29E-01

Signaling pathways	Predicted activation group	Predicted activation state	Activation z-score	P-value of overlap
FGF21	Proliferating cells (vs non proliferating cells)	Activation	3.647	3.57E-01
PDGF BB	Proliferating cells (vs non proliferating cells)	Activation	1.584	2.50E-01

Signaling pathways	Predicted activation group	Predicted activation state	Activation z-score	P-value of overlap
FGFR2	Proliferating cells (vs non proliferating cells)	Activation	3.467	3.62E-03
MAPK1	Proliferating cells (vs non proliferating cells)	Activation	2.894	2.58E-01

Signaling pathways	Predicted activation group	Predicted activation state	Activation z-score	P-value of overlap
JUN	Proliferative cells (vs non proliferative cells)	Activation	2.891	3.52E-01
TGFB1	Proliferative cells (vs non proliferative cells)	Activation	3.476	2.46E-01

Signaling pathways	Predicted activation group	Predicted activation state	Activation z-score	P-value of overlap
HGF	Proliferating cells (vs non proliferating cells)	Activation	4.751	4.85E-01
VEGFA	Proliferating cells (vs non proliferating cells)	Activation	3.484	5.62E-01

Signaling pathways	Predicted activation group	Predicted activation state	Activation z-score	P-value of overlap
AREG	Proliferating cells (vs non proliferating cells)	Activation	2.758	3.44E-03
FOS	Proliferating cells (vs non proliferating cells)	Activation	2.458	1.79E-02

Supplementary figure 4. The signaling networks of the various acetylsalicylic acid -induced prominent factors are positively correlated with self-renewal capacity. The differential activation status (either activated or inhibited) of various signaling pathways, such as EGFR (GSE21618), IGFBP6 (GSE47856), M-CSF (GSE45630), PDGFRB (GSE116237), SCFR (GSE46045), or TGFß2 (GSE48990)-related genes, between proliferative cells and non-proliferative cells was analyzed using IPA software **(A-F)**.

Supplementary figure 5

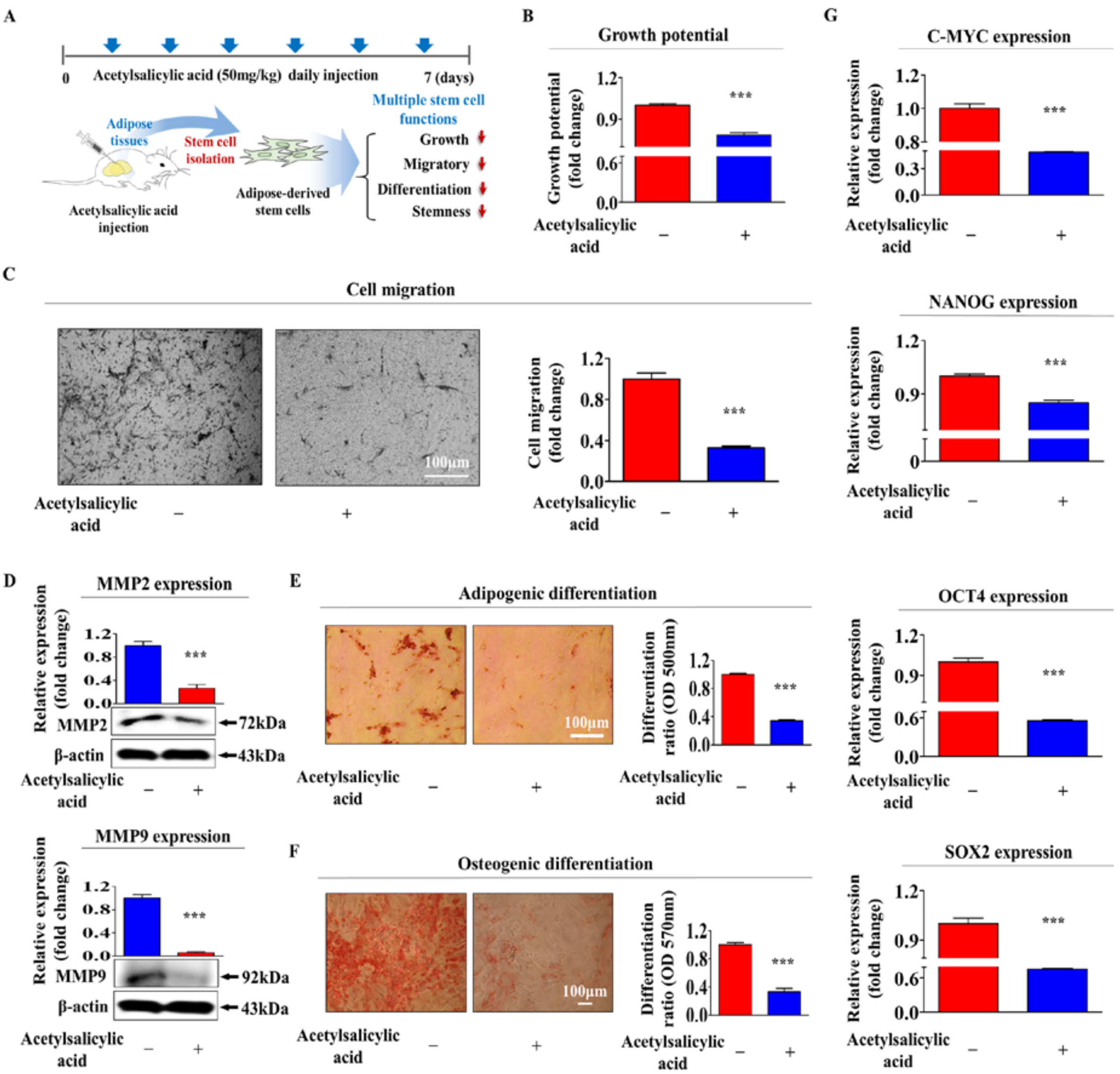

Supplementary figure 5. Acetylsalicylic acid treatment significantly inhibits various regenerative capacity-related functions of adipose tissue-derived stem cells *in vivo*. A schematic representation of the experimental procedure as described in the Materials and Methods section is shown **(A)**. The mice were treated intravenously with acetylsalicylic acid (50 mg/kg daily for 7 consecutive days), and tissue resident stem cells were isolated from mouse adipose tissues using our primary culture technique. The isolated adipose tissue-derived stem cells were cultured *in vitro* either under continuous exposure to acetylsalicylic acid (2.5 mM) or in non-acetylsalicylic acid culture conditions to mimic the physiological environment of acetylsalicylic acid exposure *in vivo*. The

subsequent inhibition of the self-renewal capacity of mouse adipose tissue-derived stem cells was analyzed by MTT assays. The stem cell proliferation rates (%) were assessed by representing the viability of the acetylsalicylic acid-treated cells as a percentage of the viability of the vehicle-treated cells **(B)**. The acetylsalicylic acid-induced inhibition of migratory potential *in vivo* was then analyzed by Transwell migration/invasion assays **(C)** and western blotting with antibodies against MMP-2 and MMP-9 **(D)**. The acetylsalicylic acid-induced inhibition of adipogenic **(E)** and osteogenic **(F)** differentiation *in vivo* were analyzed by oil red O and alizarin red staining, respectively. The cytoplasmic calcium concentration and lipid droplet (LD) formation within differentiated cells were assessed by measuring the absorbance values of the solubilized cells at wavelengths of 500 nm and 570 nm, respectively. The acetylsalicylic acid-induced inhibition of the expression of various pluripotency/stemness markers (C-MYC, NANOG, OCT4, and SOX2) *in vivo* was evaluated using real-time PCR **(G)**. β-actin was used as the internal control. HPRT was used as a housekeeping gene for real-time PCR analysis. Significant differences are presented. *$p < 0.05$, **$p < 0.005$, and ***$p < 0.001$ (One-way ANOVA).

Supplementary figure 6

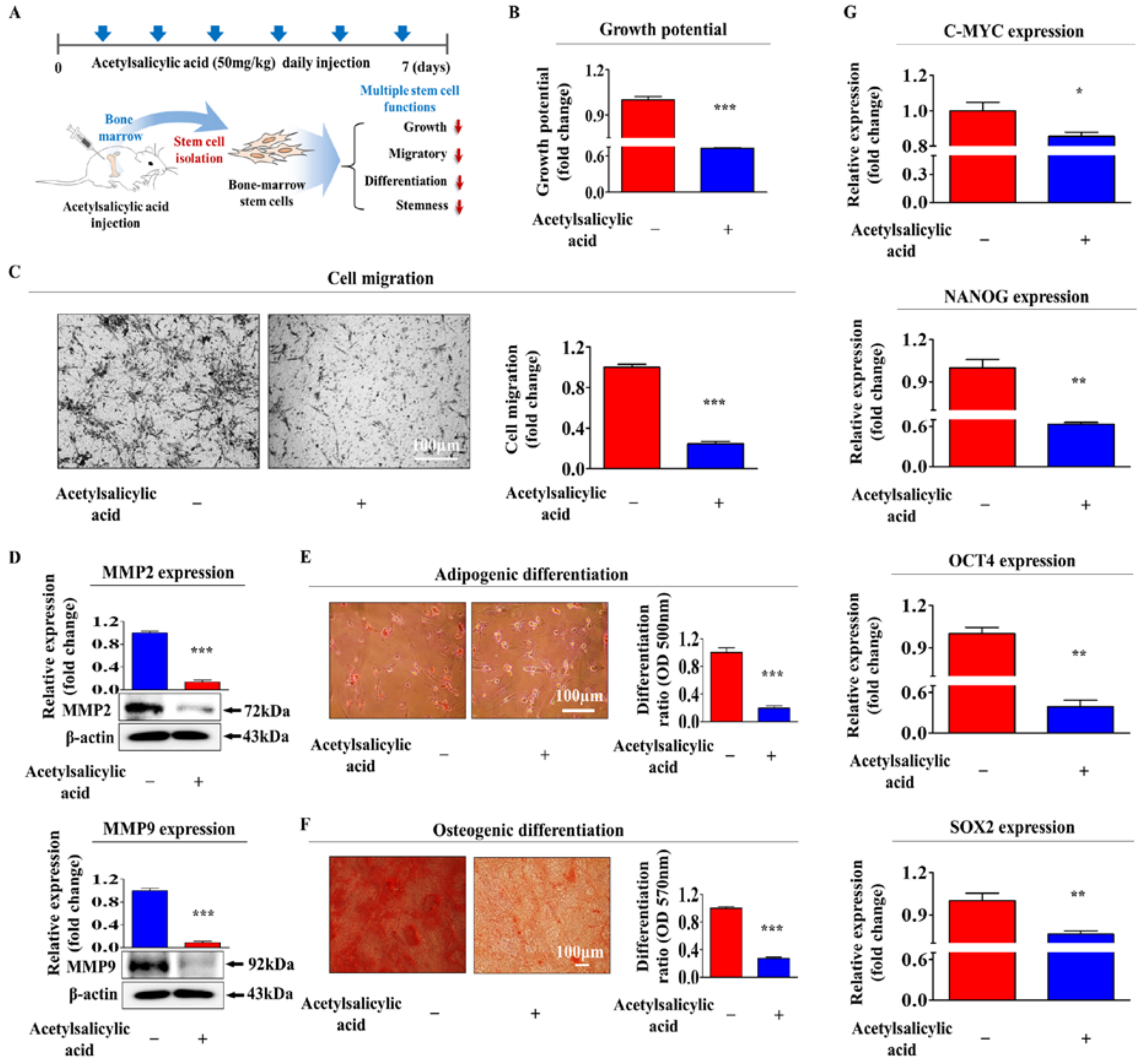

Supplementary figure 6. Acetylsalicylic acid treatment significantly inhibits various regenerative capacity-related functions of bone marrow stem cells *in vivo*. A schematic representation of the experimental procedure as described in the Materials and Methods section is shown **(A)**. The mice were treated intravenously with acetylsalicylic acid (50 mg/kg daily for 7 consecutive days), and tissue resident stem cells were isolated from mouse bone marrow using our primary culture technique. The isolated bone marrow stem cells were cultured *in vitro* either under continuous exposure to acetylsalicylic acid (2.5 mM) or in non-acetylsalicylic acid culture conditions to mimic the physiological environment of acetylsalicylic acid exposure *in vivo*. The subsequent inhibition of the self-renewal capacity of mouse bone marrow stem cells stem cells was analyzed by

MTT assays. The stem cell proliferation rates (%) were assessed by representing the viability of the acetylsalicylic acid-treated cells as a percentage of the viability of the vehicle-treated cells **(B)**. The acetylsalicylic acid-induced inhibition of migratory potential *in vivo* was then analyzed by Transwell migration/invasion assays **(C)** and western blotting with antibodies against MMP-2 and MMP-9 **(D)**. The acetylsalicylic acid-induced inhibition of adipogenic **(E)** and osteogenic **(F)** differentiation *in vivo* were analyzed by oil red O and alizarin red staining, respectively. The cytoplasmic calcium concentration and lipid droplet (LD) formation within differentiated cells were assessed by measuring the absorbance values of the solubilized cells at wavelengths of 500 nm and 570 nm, respectively. The acetylsalicylic acid-induced inhibition of the expression of various pluripotency/stemness markers (C-MYC, NANOG, OCT4, and SOX2) *in vivo* was evaluated using real-time PCR **(G)**. β-actin was used as the internal control. HPRT was used as a housekeeping gene for real-time PCR analysis. Significant differences are presented. *$p < 0.05$, **$p < 0.005$, and ***$p < 0.001$ (One-way ANOVA).
